
JOURNAL INFORMATION
==============================
NLM Title Abbreviation: J Child Orthop
Journal ID: J Child Orthop
NLM Journal ID: 101313582

Journal of Children's Orthopaedics

ISSN: 1863-2521
EISSN: 1863-2548
Publisher: SAGE Publications

ARTICLE INFORMATION
==============================
PMCID: PMC3955429
PMID: 24634289
DOI: 10.1007/s11832-014-0573-4
Article ID: 573
Article version: 1
Subjects: Abstracts

EPOS 33rd Annual Meeting

Electronic publication date: 2014 Mar 16
Print publication date: 2014 Apr
Volume: 8
Issue: Suppl 1
First page: 7
Last page: 54
Copyright: © The Author(s) 2014
License: The abstracts are distributed under the terms of the Creative Commons Attribution License which permits any use, distribution, and reproduction in any medium, provided the original author(s) and the source are credited.
License URL: https://creativecommons.org/licenses/by/4.0/

issue-copyright-statement: © The Author(s) 2014

==============================

**April 3rd**
Trauma—Upper Extremity
08:00–09:00
CONCERT HALL
**OP1**/08:00–08:10

## Evidence into practice: paediatric orthopaedic surgeons use of removable splints for common pediatric fractures

**Unni G. Narayanan**[1], Andrew Howard[1], Anna Cuomo[2]

[1]The Hospital for Sick Children, Toronto, Canada;
[2]Shriners Hospital for Children Los Angeles, Los Angeles, USA

**LEVEL 2/Trauma—Upper limb**
Keywords: Evidence based medicine, Randomized trials, Common pediatric fractures, Splints, Clinical practice

*Purpose*: Randomized trials have shown that removable splints are as safe and effective as circumferential casts for the management of many common types of paediatric wrist and ankle fractures, and often more cost-effective. This study estimated the extent to which this evidence is being implemented in clinical practice, and determined the perceived barriers to the adoption of this evidence.
*Methods*: A cross-sectional survey of practicing orthopaedic surgeon members of the Pediatric Orthopedic Surgeons of North America (POSNA) was conducted, using a 22 item online questionnaire, and distributed using a modified Dillman technique. Survey questions were derived from and validated by literature review, expert opinion, and pilot-testing on the targeted sample prior to implementation.
*Results*: Of the 826 eligible participants, 558 (67.6 %) responded to the survey. Of these, 505 (90.5 %) had completed a fellowship in paediatric orthopedics, 335 (60.0 %) worked in a university-affiliated setting, and 377 (67.6 %) had been in practice less than 20 years. Only 158/543 (29.1 %, 95 % CI 25.28, 32.92) reported using a removable splint to treat buckle fractures of the distal radius; while only 32 (5.9 %; 95 % CI 3.9, 7.9) and 8 (1.5 %; 95 % CI 0.5, 2.5) would use such splints for minimally displaced greenstick and transverse fractures of the distal radius, respectively. For distal fibular avulsion fractures, only 122 (22.5 %, 95 % CI 19.0, 26.0) would use a removable splint, while only 57 (10.5 %; 95 % CI 7.9, 13.1) and 28 (5.6 %, 95 % CI 3.7, 7.5) would do so for undisplaced Salter-Harris I and II fractures of the distal fibula, respectively. The most commonly reported perceived barriers to application of a removable device were concerns about patient compliance, potential complications, and possible medico-legal implications.
*Conclusions*: Although removable splints have been shown in randomized trials to be safe and cost-effective for the management of minor and stable fractures of the wrist and ankle, only a relatively small proportion of practicing POSNA members use such splints for these injuries. These data support the need for implementation of knowledge translation strategies to encourage paediatric orthopaedic surgeons to change practice in keeping with the best evidence for these common and stable injuries.
*Significance*: Despite level 1 evidence from randomized trials, additional steps are necessary to translate this evidence into clinical practice.

**OP2**/08:10–08:20

## Change in gap index to assess adequacy of plaster cast on follow-up radiographs and its correlation in predicting fracture stability

**Madhu Tiruveedhula**, Suri Gudipati, Brian Scott

Leeds General Infirmary, Leed, UK

**LEVEL 4/Trauma—Upper limb**

*Purpose*: To investigate whether a change in the gap index measured on follow-up radiographs following closed reduction of a distal forearm fracture predicts reduction of swelling thereby increasing the risk of re-displacement.
*Methods*: Displaced traumatic fractures of distal radius and ulna managed by closed reduction and above elbow moulded cast were included in the study. Two authors independently assessed change in the cast index and Gap index on a 2 week follow-up radiographs and compared it with intra-operative views.
*Results*: Sixty-five children with a mean age of 9 years (range 4–15 years) admitted between Jan 2008 and Feb 2010 were included in the study. Gradual loss of reduction was noted in 54 (83 %) children and 27 (41 %) of these children needed revision surgery.

As the plaster cast was not changed during this short follow-up, the cast index remained same. However, there was a significant change in the gap index in patients needing revision surgery from 0.14 on intra-operative radiographs to 0.26 at 2 weeks ($p = 0.0092$).
*Conclusions*: Maintenance of fracture reduction relies on a snuggly fitting plaster cast. But, as the swelling subsides this stabilizing affect decreases thereby risking fracture reduction. The Gap index is sensitive to this change and can be reliably used to assess loss of swelling and the plaster cast replaced before fracture reduction is lost.
*Significance*: Loss of swelling during follow-up can destabilise a well reduced fracture. Change in the gap index on follow-up X-rays has been shown in this study to reliably signify loss of swelling.

**OP3**/08:20–08:30

## Management of pulseless supracondylar fractures of the humerus in children: towards the end of the controversy?

**Adeline Cambon-Binder**[1], Pascal Jehanno[2], Laurent Tribout[2], Brice Ilharreborde[2], Philippe Valenti[2], Keyvan Mazda[2]

[1]European Georges Pompidou Hospital, Paris, France; [2]Robert Debré Hospital, Paris, France

**LEVEL 4/Trauma—Upper limb**

Keywords: Fracture, Supracondylar, Humerus, Child, Compartment Syndrome, Volkmann Contracture, Ischemia

*Purpose*: Management of supracondylar fractures in the presence of a pulseless, yet well-perfused hand remains controversial. The purpose of this study was to analyze the outcomes of a conservative treatment.
*Methods*: We reviewed a continuous series of 742 cases of displaced supracondylar fractures of the humerus operated between 1999 and 2009 to identify pulseless patients at presentation. The pre-operative data recorded were the age of the patient, the type of displacement according to the Lagrange and Rigault classification, ischemia signs (white and pulseless hand with lengthening of the recoloration duration). The limb perfusion was reassessed after reduction and pinning of the fracture. We did not explore the brachial artery in the absence of residual ischaemic signs. Early postoperative data collected were the lack of the pulses and signs of compartment syndrome. At an average of 3 years post injury (range, 4 months to 12 years), clinical examination assessing functional outcome and signs of chronic vascular impairment was carried out. Nine of the patients had a Doppler-assisted ultrasound of the injured limb. In six patients we calculated the digital-brachial index after photopletysmographic measurement of the digital arterial blood pressure.
*Results*: Twenty-eight patients showed a pulseless hand on arrival (3.8 %). All of them had a displaced extension-type fracture at stage IV of the Lagrange and Rigault classification. Three of the ten patients with pre-operative ischemia had a brachial artery bypass: one case in emergency for a wide open fracture, and later in two cases with compartment syndromes and signs of hypo-perfusion, which remained pulseless after reduction. One of these had delayed diagnosis and developed a Volkmann contracture. Patients with preoperative ischemia and palpable pulses after reduction ($n = 5$) had always satisfactory outcomes. So did patients who had an early arterial exploration for remaining ischemia after reduction ($n = 2$), and patients with a well-perfused hand at presentation ($n = 18$), whether pulses were palpable or not after reduction. No limb discrepancy, or arterial claudication were observed. Initial nerve impairment and open fractures were more at risk for vascular repair (respectively $p = 0.002$ and $p = 0.03$).
*Conclusions*: Close monitoring of patients with a well-perfused hand after reduction gave good results, whether pulses were recovered or not. We recommend a surgical exploration of the artery only in cases of remaining or recurrent ischaemic signs after reduction. High-risk patients were those with an ischaemic hand at presentation, pink but remaining pulseless after reduction. A Doppler perfusion study of the forearms would allow identifying those needing surgical repair of the brachial artery due to insufficient collateral circulation.
*Significance*: Prognostic and therapeutic.

**OP4**/08:30–08:40

## A prospective comparative study of pin tract infection in pediatric humeral supracondylar fractures. Daily pin care vs. No pin care

**Chia Hsieh Chang**, Wei Chun Li, Hsiun Kai Kao, Mei Chun Chen

Chang Gung Memorial Hospital, Taipei, Taiwan

**LEVEL 2/Trauma—Upper limb**
Keywords: Pin care, Humeral supracondylar fracture

*Purpose*: Percutaneous pinning is a common treatment in paediatric fractures. The methods of pin care are variable and controversial. This study is to compare the infection rate between daily pin care and no pin care.
*Methods*: 62 children with humeral supracondylar fracture treated by closed reduction and pinning were collected prospectively from June 2011 to March 2013. They were randomized by the arriving date. The paediatric orthopaedic surgeons preferred casting without pin care. The orthopaedic trauma surgeons preferred thermoplastic splinting and cleaning the pin tract with alcohol daily. The two teams shared the emergency duty on alternative dates. A nurse instructed in post-operative care, checked the pin site fortnightly until pin removal. Pin tract infection was graded by Moore and Dahl's 6-grade classification. The study was approved by the institutional review board.
*Results*: Each group included 31 patients. All had closed reduction and pinning on the day or next morning of arrival. Fractures united well in 6 weeks. Age, sex, fracture side, Gartland type, number of pins, and days to pins removal were comparable between the two groups. Pin tract infection grade 2 (serous discharge) and beyond were noted in 5 cases (16 %) in no pin care group, and in 16 cases (52 %) in daily pin care group ($p < 0.05$).
*Conclusions*: Daily pin care in pediatric elbow fractures had greater infection rate and parental anxiety evident by more telephone consultations.
*Significance*: Pin care after percutaneous pinning for elbow fracture fixation in children is not recommended.

**OP5**/08:40–08:50

## Pediatric supracondylar humeral fractures: is there any relation between the pinning technique and the clinical—radiological final result?

**Maria De Los Angeles De La Red Gallego**[1], Ana Alfonso Fernandez[2], Maria Dolores Garcia Alfaro[1], Tamara Rodriguez Lopez[1], Ana Ferrer Frias[2], Eduardo Saenz Medrano[2], Carmelo Arbona Jimenez[1], Maria Isabel Perez Nuñez[1]

[1]Hospital Universitario Marqués de Valdecilla, Santander, Spain; [2]Hospital Universitario de Alava-Txagorritxu, Alava, Spain

**LEVEL 4/Trauma—Upper limb**
Keywords: Pediatric, Elbow, Fracture, Supracondylar, Pinning, Percutaneous

*Purpose*: Supracondylar humeral fractures are the most common fractures in children. If they are treated improperly, there may be serious complications. The gold standard nowadays is closed reduction and stabilization with percutaneous Kirschner wires. What is still being discussed is the optimal K wires configuration.

Recently, some mechanical studies had been published, analysing the ideal configuration to obtain the maximal stability.

We started a retrospective study to try to understand if the treatment with different K wires configurations is related to the clinical and radiological results. We think that there are more factors like time in the cast, position of the arm in the cast, and doing good clinical and radiological revisions, which may influence the result.

The object of the study is to see if different configurations of the K wires really determine the final clinical and radiological result.

*Methods*: We reviewed all the patients aged between 2 and 12, treated surgically for supracondylar fractures of the humerus in our department from 1997 to 2011 (follow up at least 2 years). 195 patients were finally included. Clinical and radiological data were recorded pre- and post-operatively. The relation between the k-wire configuration and the malreductions, malunion, range of motion and varus—valgus deformities were analysed. If the lateral configuration was performed, we also recorded the angle between the two k—wires in the AP projection, to know if the divergency between the K wires is important to the final result.

*Results*: The most common configuration used was 2 lateral K wires (66.3 %), with an average of 16° divergent. The cast was maintained around 5 weeks. 16 patients were re-operated, 50 % were initially treated with this K wire disposition (no statistically differences were found). The final Baumann angle was not in relationship with K-wire configuration. 91 % of the patients had an excellent result regarding the Flynn test.

*Conclusions*: Closed reduction and percutaneous pinning is the right treatment for childhood displaced supracondylar fractures. What is still in debate is the optimal configuration of the K wires to be strong enough to maintain the reduction. Use of a medial pin is controversial because the risk of neurological complications.

With the results obtained in our study, the pin configuration is not so important as is stated in mechanical studies. Achieving a good reduction before the pin insertion and a close clinical monitoring until the fracture heals is essential to get an excellent result.

*Significance*: This study includes more than 180 patients, with at least 2 years of follow up. Our case series is one of largest published to our knowledge. The conclusions of this study suggest that the configuration of the K wire is not important to the final result after a supracondylar fracture.

**OP6**/08:50–09:00

## Neurological and vascular injury associated with supracondylar fractures of the humerus and ipsilateral forearm fractures in children

**Robert Lane Wimberly**[1], Anthony Ian Riccio[1], Ryan D Muchow[2], Christine A Ho[1], Sumeet Garg[3]

[1]Texas Scottish Rite Hospital for Children, Dallas, USA; [2]University of Kentucky, Lexington, USA; [3]Unviersity of Colorado, Denver, USA

**LEVEL 3/Trauma—Upper limb**

Keywords: Supracondylar humerus, Ipsilateral, Floating elbow

*Purpose*: Approximately 5 % of supracondylar humerus fractures in children are associated with an ipsilateral forearm fracture, often referred to as a floating elbow injury. Historically, these patients have higher complication rates than patients with an isolated supracondylar fracture of the humerus. Our purpose is to review the acute neurological and vascular injuries in patients with ipsilateral, operative supracondylar humerus and forearm fractures and compare the findings with a cohort of isolated, operative supracondylar fractures of the humerus.

*Methods*: We performed an IRB approved, retrospective review of all pediatric patients with ipsilateral, operative supracondylar fractures of the humerus and forearm fractures from a single institution and compared our findings to a cohort of isolated, operative supracondylar fractures of the humerus.

*Results*: 150 patients with ipsilateral supracondylar fractures of the humerus and forearm fractures were compared to 1228 patients with isolated, surgically managed supracondylar fractures. 22 of 150 (14.7 %) floating elbow patients had documented pre-treatment nerve palsies compared to 96/1228 (7.8 %) of isolated injury patients ($p = 0.006$). 18/22 nerve palsies were in patients with forearm fractures that required reduction. The overall incidence of nerve palsy was 18.9 % (18/95) when a forearm fracture required reduction compared to only 7.3 % (4/55) in a forearm fracture that was not reduced ($p = 0.05$). We did not find a significant difference in the rate of pulseless extremities when comparing the ipsilateral (6/150 4 %) and isolated (50/1228 4.1 %) injury patients. No compartment syndromes were identified in any patient with an ipsilateral injury.

*Conclusions*: The rate of acute neurological injury in ipsilateral supracondylar and forearm fractures is almost twice that found in patients with isolated supracondylar fractures. This rate increases further when the forearm fracture requires a manipulative reduction. The likelihood of a pulseless extremity was not dependent upon the presence of a forearm injury in our study. The presence of an ipsilateral forearm fracture should alert the surgeon carefully to assess the pre-operative neurovascular status of patients with supracondylar injuries.

*Significance*: Ipsilateral forearm fractures associated with a supracondylar fracture of the humerus are indicative of higher likelihood of acute neurological injury. With more displaced forearm fractures, the incidence of nerve injuries rises.

**April 3rd**
Clinical Science—Prize Papers
09:00–10:00
CONCERT HALL
**OP7**/09:00–09:10

### Supracondylar fractures with isolated anterior interosseous nerve injuries: are they urgent cases?

**Kody K. Barrett**[1], David L. Skaggs[1], Jeffrey R. Sawyer[2], Lindsay Andras[1], Alice Moisan[2], Christine Goodbody[3], Jack Flynn[3]

[1]Children's Hospital, Los Angeles, United States; [2]Campbell Clinic, Germantown, United States; [3]Children's Hospital, Philadelphia, United States

**LEVEL 3/Trauma—Upper limb**
Keywords: Supracondylar fracture, Nerve palsy

*Purpose*: To determine if patients with isolated anterior interosseous nerve (AIN) injuries and supracondylar fractures of the humerus (SCH) should be treated urgently.
*Methods*: A retrospective multi-center study of 5,546 patients with operative supracondylar humerus fractures was conducted. Exclusion criteria were nerve injuries other than the AIN, pulselessness, associated forearm fracture, open fractures, $< 2$ months follow-up, or pathological fractures.
*Results*: Thirty-six patients met inclusion criteria. The average time to surgery in these cases was 14.6 h (range 2–36). No patients developed a compartment syndrome or any other complications from the injury or surgery. There was no statistically significant difference in time to total return of AIN function when stratified by time to surgery (0–6 h = 54.25 days, 6–12 h = 53 days, 12–24 h = 74.0 days; $P = 0.668$). Complete return of AIN function occurred in all patients with an average time to complete return of 65 days (range 2–224).
*Conclusions*: This is the largest series of AIN injuries with SCH fractures in the literature. There is no evidence in this series that an isolated AIN injury in the setting of a SCH fracture requires the fracture to be treated urgently. Delay in treatment up to 24 h did not lengthen the time of nerve recovery or lead to other complications.

It is critically important to stress that this series excluded cases with pulselessness, other nerve injuries, and associated forearm fractures which all may require urgent surgery, and that surgeons may have treated other cases urgently based on swelling, ante-cubital ecchymosis or other factors suggesting urgent care was needed.
*Significance*: Barring other clinical indications for urgent treatment of a SCH fracture, an isolated AIN may not by itself be an indication for urgent surgery. All AIN injuries in this series showed complete recovery at a mean of 65 days.

**OP8**/09:10–09:20

### An anatomical study of the greater trochanter starting point for intramedullary nailing

**Kathleen Farhang**[1], Ronak Desai[1], John H. Wilber[1], Daniel R. Cooperman[2], Raymond W. Liu[1]

[1]Case Western Reserve University, Cleveland, USA; [2]Yale University School of Medicine, New Haven, USA

**LEVEL 4/Trauma—Lower limb**
Keywords: Trochanteric femoral nail, Greater trochanter, Anatomy

*Purpose*: Intramedullary nail insertion through a greater trochanter starting point has been increasingly used in the adolescent population. Although the optimal position for AP insertion has been well characterized, lateral insertion position is poorly defined.
*Methods*: 744 paired femora from well-preserved cadavers were placed both in a neutral apparent neck-shaft angle (ANSA) position, and with internal rotation to neutralize femoral anteversion in a true neck-shaft angle (TNSA) position. A marker was placed at the apex of the greater trochanter from the anterior viewpoint to simulate placement of a guidewire at the tip of the trochanter. The perpendicular distance between the marker and the centre of the intramedullary canal was measured on AP and lateral views. The angle of anteversion was measured between the bicondylar plane and femoral neck. In a subset of 276 femora, the greater trochanter morphology was graded into 4 groups: anterior lean, posterior lean, centred and flat. Multivariate Pearson product-moment correlation analysis was performed to determine the influence of morphological variance and anteversion on the accuracy of using the greater trochanteric apex as a starting point for intramedullary nail insertion.
*Results*: Mean age was $56 \pm 11$ years. Mean anterior displacement of the trochanteric apex was $5.0 \pm 4.1$ and $4.6 \pm 4.2$ mm relative to the intramedullary canal for the ANSA and TNSA positions respectively ($p < 0.0005$). Mean lateral displacement of the apex was $7.1 \pm 4.6$ mm for the ANSA view and $6.4 \pm 4.6$ mm for the TNSA view ($p < 0.0005$). In both the ANSA and TNSA views, there was a weakly positive association between anterior lean morphology and anterior displacement ($r = 0.156$, $p < 0.05$; $r = 0.173$, $p < 0.01$) and between flat morphology and lateral displacement ($r = 0.172$, $p < 0.01$; $r = 0.141$, $p < 0.05$). Anteversion negatively correlated with anterior displacement weakly in both ANSA and TNSA views ($r = -0.084$, $p < 0.05$; $r = -0.150$, $p < 0.01$).

*Conclusions*: The apex of the greater trochanter is lateral and anterior relative to the intramedullary canal, and the magnitude of lateral and anterior displacement is minimally reduced when the femur is internally rotated. Greater trochanteric morphology and femoral anteversion had statistically significant but modest effects on this relationship.
*Significance*: Trochanteric intramedullary nails are designed to compensate for lateral displacement of the greater trochanteric apex, but not for anterior displacement. A starting point on the tip of the trochanter in the AP view does not reliably center in the sagittal plane. Based on this data, intramedullary nail insertion sites should be about 5 mm posterior to the trochanteric apex to account for its anterior positioning.

**OP9**/09:20–09:30

**Predictive significance of baclofen test for GMFCS level 3–4 children with cerebral palsy before selective dorsal rhizotomy**

**Vladimir Kenis**

The Turner Institute for Children's Orthopaedics, Saint-Petersburg, Russia

**LEVEL 2/Cerebral palsy**
Keywords: Cerebral palsy, Rhizotomy, Baclofen test

*Purpose*: Selective dorsal rhizotomy (SDR) is an effective procedure for spasticity in patients with upper motor neuron lesions, including cerebral palsy. The indications for surgery are controversial for children with GMFCS levels 3–4 due to unpredictable influence on motor performance even after successful decrease of the muscle tone. Unfavorable decrease of "useful spasticity" can lead to deterioration in assisted standing and walking. Intra-thecal baclofen therapy (ITB) is a more controllable tool for reduction of generalized spasticity. Prediction of the results is possible with baclofen test (BT)—single intra-thecal injection of baclofen. Disadvantages of ITB include necessity of refilling of the pump, size of device and possible adverse effect of medication. Some families reject ITB in favor of SDR.
*Aim*: To assess the significance of the baclofen test for GMFCS level 3–4 children with cerebral palsy before selective dorsal rhizotomy.
*Methods*: The whole series included 66 patients with spastic cerebral palsy aged from 5 to 9 years with GMFCS level 3–4.

In the first series of 26 consecutive patients baclofen test was done as a part of selection program for ITB therapy. In 8 patients ITB was performed in neurosurgical clinic. For the residual 18 children from this series SDR was performed in 8 children during the next 1–3 months as an alternative because of refusal of the parents from ITB (group 1).

Group 2 included 20 patients who were scheduled for SDR and underwent primarily baclofen test for preoperative planning. A Control group included 20 patients who underwent SDR without baclofen test during last 3 years.

Changes in the ability of assisted standing and walking were assessed by clinical observation and videotaping in 6 and 12 months after surgery.
*Results*: An empirical attempt to compare the results of SDR with BT which was performed before surgery demonstrated an obvious correlation: in 2 children reduction of spasticity led to worsening in standing and walking, which corresponded to a retrospective analysis of the records of the BT results.

In the Group 2 SDR was done only in 12 patients without functional impairment after BT, and functional deterioration was avoided in this group.

In retrospective control group functional deterioration due to loose of "useful spasticity" was revealed in 15 % of children.
*Conclusions*: Functional deterioration after SDR can be predicted by BT.
*Significance*: Unsatisfactory results of SDR can be avoided by preoperative planning with BT in GMFCS level 3–4 children with cerebral palsy.

**OP10**/09:30–09:40

**The Ponseti method is superior to surgical treatment in idiopathic clubfoot – prospective randomized long-term trial**

**Martin Švehlík**, Gehardt Steinwender, Ulrike Floh, Elisabeth Wolf, Tanja Kraus

Medical University Graz, Graz, Austria

**LEVEL 2/Clubfoot**
Keywords: Clubfoot, Ponseti, Surgery

*Purpose*: Treatment of idiopathic clubfoot has shifted from extensive surgical release to the conservative Ponseti method. However, randomized prospective trials to compare both methods are lacking.
*Methods*: In 2001 we began a single center, prospective, randomized, controlled trial with a parallel design. 24 feet in 15 patients were randomly divided in two groups: 12 feet belonged to the Ponseti group and 12 feet to the surgical group treated by the McKay Simons procedure. The average follow-up for the present study was 10 years. To compare the morphology, functional, radiologic and subjective outcomes between the groups, several outcome measures were used (ICFSG = International Clubfoot Study Group score, PODCI = Pediatric outcomes Data Collection Instrument and FRS = Functional Rating Score). The results were compared statistically by the Mann–Whitney U test.

*Results*: Both groups were comparable at the beginning of treatment with no significant difference in the Pirani score ($p = 0.623$). After 10 years of follow-up the Ponseti group were superior in morphology ($p = 0.024$), functional ($p = 0.006$) as well as radiological ($p = 0.023$) outcomes according to ICFSG. Also PODCI ($p = 0.016$) and FRS ($p = 0.005$) showed better functional status in the children treated with the Ponseti method. Children treated with surgery had more troubles performing sports ($p = 0.003$) and experienced more pain ($p = 0.006$), which might also led to inferior results in the happiness domain of PODCI ($p = 0.030$)

*Conclusions*: In this prospective randomized long term study the Ponseti method presented superior outcomes to surgery in the treatment of idiopathic clubfoot deformity.

*Significance*: Our study confirms that the Ponseti technique is a preferable method of treatment in idiopathic clubfoot deformity.

**OP11**/09:40–09:50

### Is a posterior only approach sufficient to restore thoracic hypokyphosis in the management of adolescent idiopathic scoliosis?

**Emilie Peltier**, Sebastien Pesenti, Benjamin Blondel, Elie Choufani, Gerard Bollini, Jean-Luc Jouve

Aix-Marseille University, Marseille, France

**LEVEL 4/Spine**

Keywords: Idiopathic scoliosis, Hypokyphosis, Hybrid construct

*Purpose*: Performing a posterior all-screws fixation for adolescent idiopathic scoliosis (AIS) can lead to a loss of thoracic kyphosis. Conversely, hybrid constructs may preserve a better sagittal alignment, especially a better restitution of thoracic kyphosis in hypokyphotic patients.

*Methods*: 29 patients with AIS were included in this single centre retrospective observational study. The surgical procedure included a systematically hybrid construct with pedicular screw fixation between T11 and the lower instrumented vertebra, sub-laminar bands in the concavity of the deformation and hooks at the upper level.

Radiographic measurements were performed pre- and post-operatively including Cobb angle, regional sagittal parameters (cervical lordosis, thoracic kyphosis and lumbar lordosis) and pelvic parameters (Pelvic Incidence, Pelvic tilt, Sacral Slope).

*Results*: For the whole series, a significant reduction of Cobb angle was reported with an average correction of 70 % (58.1° vs. 16.9°, $p < 0.001$).

Modification of the thoracic kyphosis was not correlated to the Cobb angle correction ($r = 0.085$, $p = 0.339$), but was significantly correlated to the changes in cervical alignment ($r = 0.354$, $p = 0.045$).

*Conclusions*: A posterior only approach using a hybrid construct in the management of AIS leads to a good correction of hypokyphotic patients. This correction of the thoracic kyphosis is responsible for a cervical reciprocal change with reappearance of a cervical lordosis. This posterior only surgical strategy leads to a restitution of sagittal alignment associated with a satisfactory coronal correction of the deformity. Therefore, performing a prior anterior discectomy does not seem necessary in the management of hypokyphotic AIS patients. Further studies with larger cohort and longer follow-up will be needed in order to confirm these results.

*Significance*: A hybrid construct allows a restoration of thoracic kyphosis in hypokyphotic patients. These changes are responsible for sagittal reciprocal changes in the cervical lordosis.

**OP12**/09:50–10:00

### Characterisation of hip morphology in children with Mucopolysaccharidosis Type I/II

**Markus Baker**, Elizabeth Ashby, Deborah Eastwood

Great Ormond Street for Children, London, UK

**LEVEL 4/Tumors and metabolic disorders**

Keywords: Hip morphology Mucopolysaccharidosis

*Purpose*: The purpose of this study is to characterise the prevalence, morphology and natural progression of hip disease in children with mucopolysaccharidosis Type I/II.

*Methods*: This is a retrospective case series report of the hip anatomy of children with MPS Type I/II at a UK teaching hospital from 2004 to 2013. Hip X-rays were examined to determine Reimer's index (as a measure of hip subluxation) and the shape of the femoral head. Children had an average of 4 hip X-rays with an interval of between 9 and 24 months between images.

26 patients had an X-ray performed of both hips at the age of 8. Reimer's index is reported at this age to give a 'snap-shot' of femoral head coverage. To assess head morphology, images were converted into digital line drawings and then converted to composite images. Deconvolution was used to create a single 'average' image. This image was then overlaid onto a normal matched hip to define and describe the morphological differences.

24 patients had a minimum of 3 hip X-rays over a 5-year period. The change in Reimer's index is reported for these patients to assess disease progression.

*Results*: 52 hips in 26 patients age 8 years were radiographically analysed. 2 hips showed no evidence of subluxation. 45 hips were subluxed: 14 were mildly subluxed (Reimer's index 1–20), 10 hips were moderately subluxed (Reimer's Index 21–50) and 23 hips were severely subluxed (Reimer's Index 51–99). 3 hips were dislocated.

48 hips in 24 patients were radiographically analysed over a 5-year period: 30 % deteriorated significantly over this time leading to dislocation and 70 % remained stable.

Image analysis using deconvolution revealed relative preservation of the metaphyseal region and lateral epiphyseal region, whilst there was proportionally greater disruption of the medial epiphysis.

*Conclusions*: Children with MPS Type I/II have marked bony uncovering of the femoral head. Approximately one third of these hips progress to dislocation but two-thirds remain relatively stable and do not sublux further. With regards to femoral head morphology, there appears to be relative preservation of the lateral epiphysis but gradual disruption of the medial epiphysis with time.

*Significance*: With the evolution of new medical treatments for children with mucopolysaccharidosis Type I/II, both life expectancy and quality of life are increasing. This study shows that almost one third of hips in these children will progressively sublux and eventually dislocate. Therefore these children should be under active surveillance and surgical hip intervention should be offered if appropriate.

**April 3rd**
Basic Science—Prize Papers
10:30–11:30
CONCERT HALL
**OP13**/10:30–10:40

### Transphyseal sliding flexible intramedullary nailing and residual growth: An experimental study

**Dominique Barbier**[1], Natalia Kononovich[2], Roman Shutov[2], Pierre Journeau[1], Dmitry Popkov[2]

[1]Service d'orthopédie pédiatrique, Hôpital d'Enfants, CHU de Nancy, Vandoeuvre-Lès-Nancy, France, Metropolitan; [2]The Federal State-Financed Institution « Russian Ilizarov Scientific Center for Restorative Traumatology and Orthopaedics » , Kurgan, Russia

**LEVEL 2/Basic science**

Keywords: Growth, Flexible intramedullary nailing, Transphyseal

*Purpose*: Transphyseal Sliding Flexible Intramedullary Nailing (TS-FIN) has been used in young children with osteogenesis imperfecta since Métaizeau in 1987 in order to reduce the risk of deformities. However, the effects of TS-FIN on residual growth are unknown. The aim of this experimental study was to examine the possibility of tibial growth disturbances caused by TS-FIN in immature dogs.

*Methods*: Two 1.8 mm stainless steel nails with opposing curves were used in six 6-month-old dogs. One was hand pushed into the medullary canal through the central area of the proximal tibial epiphysis, the second through the distal epiphysis in the region the medial malleolus. The entry hole was created with an awl. Surgery was performed on the right limb only, while the contralateral limb served as control. Radiological parameters such as length of the tibia, external and medullary midshaft diameters and cortical index, proximal medial and distal lateral angles were measured monthly on both sides on radiographs for each dog until the end of growth by 3 observers.

*Results*: There was no significant inter-observer difference in all measurements. Neither infection nor neurological complications nor slide failure were observed. Duration of growth ranged from 3 to 5 months. At the end of growth, the operated tibia became shorter in 4 specimens. The length difference was statistically significant as compared to the control limbs. The shortening ranged from 2 to 6 mm. The proximal and distal tibial angles were unchanged at the end of growth in operated bones while an average 3.2° increase of the proximal angle and a 3.3° decrease of the distal angle appeared in the control side. The medullary diameter of the operated tibia was narrower with a statistically significant higher cortical index (57 % versus 39 %).

*Conclusions*: Endomedullary and periosteal vascularisation has been reported to be increased by FIN (Teot 1987; Lascombes, 2006). This tendency of reduced medullary diameter and increased cortical index can be caused by an endosteal reaction induced by intramedullary nails and their slow movements during growth. TS-FIN seems to affect the residual growth by preventing the natural tibial angular changes during the growth of dog. Biomechanics of this phenomenon is unknown. We suggest one or more episodes of epiphysiodesis due to partial transphyseal passage of nails causing irregular longitudinal growth.

*Significance*: Transphyseal Sliding Flexible Intramedullary Nailing increases the cortical index by stimulation of endosteal reaction. Its impact on growth is slight but should be considered. The biomechanics of this disturbance is still to be discovered.

**OP14**/10:40–10:50

## Physeal morphology after thermal epiphysiodesis using RFA

**Juan Manuel Shiguetomi-Medina**, Ole Rahbek, Bjarne Møller-Madsen

Aarhus University Hospital, Aarhus, Denmark

**LEVEL 1/Basic science**

Keywords: Radiofrequency ablation, Epiphysiodesis, Physeal morphology, Histology

*Purpose*: Thermal epiphysiodesis using RFA can disrupt the growth plate (physis) morphology without damaging the adjacent articular cartilage. This study describes the macro and micro morphological changes after this procedure.
*Methods*: Epiphysiodesis using RFA was undertaken in vivo for 8 min (92–98 °C) in two ablation sites (medial and lateral) in one randomly-selected tibia in the tibia physis of 8 growing pigs. The contralateral tibia was left intact and was used as control. The pigs were followed for 12 weeks. During this period, they had free access to food and water and were able to move freely. At the end of the study, the pigs were terminated and the tibiae harvested. The specimens were studied macroscopically and histology samples were obtained. The specimens were embedded in methylmetacrylate and 7 μm sections were stained with toluidine blue and H&E. Physeal morphology, thickness and characteristics were then described.
*Results*: Macroscopically, the articular cartilage was normal in all the treated tibiae. The entrance holes for the RFA probe could be identified. In coronal sections, the physis was detected as a discontinuous line on the treated tibiae while it was seen as a continuous line in the control tibiae.

In histological sections from controls, the mean thickness of the physis was 625 μm (606–639, SD = 14). Control sections were void of disorganized structure in the different layers of the physis. In sections from the treated physis, disorganized layers in a heterogeneous line were observed. Further, bone bridges were identified at the ablation sites. The central part of the physis looked normal. Next to the bone bridge, the physis was thicker and presented fibrosis. The mean thickness of the treated physis was 820 μm (628–949, SD = 130). No abnormalities in the articular cartilage were observed.
*Conclusions*: Thermal epiphysiodesis with RFA disrupts the physeal morphology and causes the formation of bone bridges at the ablation sites. This procedure does not damage the adjacent articular cartilage. The damaged tissue, besides the bone bridges, is characterized by a disorganization in the structures of the physis and the presence of fibrosis.
*Significance*: Epiphysiodesis is the most common procedure to treat LLD. RFA is a minimal-invasive technique that can achieve epiphysiodesis safely and overcome surgery-related possible complications.

**OP15**/10:50–11:00

## Biomechanical comparison of fatigue and load-bearing performance of locked and unlocked elastic stable intramedullary nailing

Johannes Eichler[1], Leopold Berger[2], **Stefan Fischerauer**[1], Barbara Weiss[2], Anna Celarek[2], Christoph Castellani[1], Annelie Weinberg[1], Elmar Tschegg[2]

[1]Medical University of Graz, Graz, Austria; [2]Vienna University of Technology, Vienna, Austria

**LEVEL 1/Basic science**

Keywords: Elastic stable intramedullary nailing, Paediatric long bone fracture

*Purpose*: Elastic stable intramedullary nailing (ESIN) is a very common method for the treatment of paediatric long bone fractures. Due to the fact that ESIN nails offer the chance of micro motion during the healing process, this method is beneficial in comparison to rigid bone fixation and stimulates the formation of a callus [1]. The time between the incident of the fracture and complete formation of the stabilizing callus seems to be a critical phase for the implant's load-bearing. Torsional and axial stability has to be ensured by the ESIN implant during this phase. Several types of ESIN Nails underwent biomechanical observation of the working forces in this study, using different types of locking nails. The studies [2] and [3] show an increased failure rate in adult long bones after 5000 cycles, compared to other fixation techniques.
*Methods*: Due to the study aim of monitoring the period until the formation of a callus, sheep cadaver tibiae (3–4 months old) were implanted after osteotomy at the mid diaphyseal region. Four different combinations of locking systems and ESIN implants were observed during this study. Synthes TEN Titanium with endcaps (n = 7), Hofer Medical HSNesin Titanium unlocked (n = 8), Hofer Medical STEN Steel with eye and 3 mm screw (n = 8) and Hofer Medical HSNesin Titanium with plug and 3 mm screw (n = 8) were used. All nails were 3 mm in diameter.

Cyclic mechanical loading was applied using a commercial uniaxial testing device (1710DLL-5KN, Dynamess, Germany) and a pneumatic torsion testing module which was constructed by one of the authors. This device is able to apply axial load and torque to the specimen simultaneously.
*Results*: Juvenile sheep bones were used in this study to generate similar conditions to paediatric long bones. All samples failed by a closure of the initial osteotomy gap of 10 mm. The results of biomechanical tests showed significantly higher load bearing capability with each interlocking system than with the unlocked ESIN. (1000 N max. compared to 200 N). The unlocked system and the endcap ESIN failed very abruptly, while the 3 mm plug and the steel system failed slowly. Above all the 3 mm plug with steel ESIN experienced gap closure without any damage to plugs or screws, which led to a distal penetration of the diaphysis by the nails.
*Conclusions*: Interlocking systems seem to be beneficial for stability of ESIN nailing under cyclic and simultaneous axial and torsional loading. The strongest combinations in this study were Hofer steel nails and Hofer plugs with 3 mm locking screws.
*Significance*: Different combinations of ESIN nails and interlocking systems show diverse load bearing behaviors. Desirable characteristics of non-abrupt failure during the nail's loading and maximal strength of interlocking systems could be established.
*References*:

[1] Bishop NE, van Rhijn M, Tami I, Corveleijn R, Schneider E, Ito K. Shear does not necessarily inhibit bone healing. Clin Orthop Relat Res 443.

[2] Castellani C. Biomechanische untersuchung unterschiedlicher osteosyn- thesen bei dislozierter suprakondyl̈arer humerusfraktur im kindesalter, Ph.D. thesis, Universiẗatsklinik Graz (2002).

[3] Weinberg A, Castellani C, Arzdorf M, Schneider E, Gasser B, Linke B. Osteosynthesis of supracondylar humerus fractures in children: a biomechanical comparison of four techniques. Clin Biomech. 2007;22(5):502–9.

**OP16**/11:00–11:10

## Ultra-pure magnesium alloys for use as biodegradable paediatric osteosynthetic material

**Tanja Kraus**[1], Elisabeth Martinelli[1], Stefan Fischerauer[1], Johannes Eichler[1], Peter J. Uggowitzer[2], Joerg F. Loeffler[2]

[1]Medical University, Graz, Austria; [2]ETH, Zurich, Switzerland

**LEVEL 4/Basic science**
Keywords: Biodegradable materials, Magnesium, Osteosynthesis

*Purpose*: Due to their excellent properties, magnesium (Mg) alloys are ideal candidates for the use in osteosynthesis [1]. As a second operation for implant removal can be avoided they might be of special interest in paediatric orthopaedics.

However, conventional degradation of Mg alloys is too fast and accompanied by the formation of large amounts of hydrogen gas [2]. To decelerate the corrosion rate, rare-earth elements or alloying elements of questionable toxicity have been frequently implemented limiting magnesium's current application in medicine. This study investigates a new generation of ultra-high pure (XHP) magnesium alloys, containing only biocompatible elements such as zinc (Zn) and calcium (Ca).
*Methods*: Two different ultra-high pure magnesium alloys (XHP-MgZn1Ca0.3 and XHP-MgZn1.5Ca0.25) were used. Pins of 1.6 mm in diameter and 8 mm in length were implanted transcortically in 12 male Sprague–Dawley rats ($n = 6$ per group). The degradation rate, gas amount, implant-bone interface, and new bone growth were observed within a period of 6 months by means of continuous online μCT monitoring.
*Results*: Both alloys show the desired low level of degradation. For the alloy XHP-MgZn1Ca0.3 no gas formation was clinically observable. The surrounding tissue resorbed the low amount of hydrogen gas formed during the slow degradation. The degradation rate was 'in equilibrium' with new bone growth and no adverse reaction of the surrounding bone was observed.
*Conclusions*: Ultra-high pure magnesium alloyed with well-balanced amounts of Zn and Ca exhibit a low degradation rate without formation of hydrogen gas pockets. We attribute the slow degradation to the absence of micro-galvanic corrosion.
*Significance*: This new generation of magnesium alloys is characterized by ultra-high purity and the addition of small amounts of Zn and Ca as alloying elements. These new alloys fulfil all the requirements for the clinical use and are promising as biodegradable osteosynthetic devices in paediatric orthopaedics.
*References*:

(1) Staiger MP, Pietak AM, Huadmai J, Dias G. Magnesium and its alloys as orthopedic biomaterials: a review. Biomaterials. 2006;27:1728–34.

(2) Kraus T, Fischerauer SF, Hänzi AC, Uggowitzer PJ, Löffler JF, Weinberg AM. Magnesium alloys for temporary implants in osteosynthesis: in vivo studies of their degradation and interaction with bone. Acta Biomater. 2012;8(3):1230–8.

**OP17**/11:10–11:20

## Age-dependent normal values for bony, cartilaginous and labral coverage in the pediatric hip measured on MRI

**Hanspeter Huber**[1], Laurence Mainard-Simard[2], Pierre Lascombes[3], Jean-Baptiste Meyer[2], Renaud Fay[2], Pierre Journeau[2]

[1]University Children's Hospital, Zurich, Switzerland; [2]Children's Hospital of Nancy, Nancy, France; [3]Division of Pediatric Orthopaedics, University of Geneva, Geneva, Switzerland

**LEVEL 4/Basic science**
Keywords: Hip dysplasia, MRI, Normal values

*Purpose*: MR imaging is becoming more popular in the evaluation of residual hip dysplasia. Normal values of the bony, cartilaginous and labral coverage in MRI have not been published. The aim of our study was to establish reference values for normal hips at different ages and to assess the inter-observer variability of such measurements.
*Methods*: MR images of 115 normal hips in 73 children were analysed. The bony, cartilaginous and labral acetabular index (AI bone/cartilage/labrum) were measured in the coronal plane just posterior to the inferior branch of the triradiate cartilage. In order to determine interobserver variability, measurements were made by 3 different observers. Percentile graphs were established from the Student's t-distribution of the measurements grouped by 2 years of age.
*Results*: Global interobserver variability for the measurement of the AI bone was excellent (Intraclass correlation coefficient ICC 0.88). For the AI cartilage and labrum the ICC was somewhat lower (ICC 0.78) but still rated as good. Age-dependent percentile graphs of the AI bone, cartilage and labrum are presented. Although the AI bone decreased during childhood, the AI cartilage as well as the AI labrum stay relatively constant with the 50 percentile around 5° for the AI cartilage and -5° for the AI labrum. The 90 percentile is around 10° for the AI cartilage and 0° for the AI labrum
*Conclusions*: We present percentile graphs of age-related normal values. Although bony coverage increases during childhood, cartilaginous and labral coverage stay constant. We think that an AI cartilage above 10° or an AI labrum above 0° might be a good additional parameter to classify hips into the group of residual dysplasia.
*Significance*: The knowledge of normal values for cartilaginous and labral coverage is a valuable adjunct in decision making for secondary surgery in residual dysplasia.

**OP18/**11:20–11:30

## Identification of the injury level after spinal cord damage during spinal surgery. A new experimental method

Jesus Burgos-Flores[1], **Ignacio Sanpera Jr**[2], Javier Cervera[3], Eduardo Hevia Sierra[4], Carlos Barrios[5]

[1]Hospital Ramon y Cajal, Madrid, Spain; [2]Hospital Universitari Son Espases, Palma De Mallorca, Spain; [3]Hospital Virgen de la Salud, Toledo, Spain; [4]Clinica la Fraternidad, Madrid, Spain; [5]Universidad Católica de Valencia, Valencia, Spain

**LEVEL 2/Basic science**
Keywords: Spinal cord injury, Neuromonitoring

*Purpose*: To test a new neuro-monitoring (NMT) system to identify the level of injury occurring during surgery.
*Methods*: 5 domestic pigs were used for the study.

Bilateral laminectomies were performed at T4–T5, T7–T9 and T12–T13 segments. Pedicle screws were inserted on the left pedicle T5, T7, T9 and T12.

4 epidural catheters were placed sublaminar for NMT at the level of T3, T6 T11 and L1 (2 above and 2 below the injury).

And the NMT was done using: (1) cord–cord motor evoked potential (MEP) between epidural catheters, (2) Sensory epidural potentials (SEP) after stimulation of a mixed nerve in the lower limb, (3) recording of the motor D-wave after transcranial stimulation and (4) Pulse-train stimulation of the screws inserted recording response in the epidural catheters.

After recording potentials a section of the spinal cord at the level of T8 was performed and potentials were registered again.
*Results*: After cord section, the following recording was observed:

(1) Lack of cord–cord MEP between catheters placed caudal and cranial to the lesion.
(2) SEP were normal below the injury level and normal above.
(3) Motor D-wave was normal above the injury, absent below.
(4) Pulse-Train stimulation of screws above the spinal cord section showed caudal response distal to the lesion in 3 occasions.

*Conclusions*: Intra-operative identification of the level of spinal cord injury is feasible by an NMT system. The pulse train screw stimulation was the less reliable technique.
*Significance*: When spinal cord injury occurs during surgery, early identification of the spinal level is crucial to try to lessen the damage. This NMT protocol shows that is possible to locate the level of injury during surgery.

**April 3rd**
Club-Foot
11:30–11:40
CONCERT HALL
**OP19/**11:30–11:40

## Correlation of gait analysis and radiographic measurements in clubfoot treated with the Ponseti method

**Christof Radler**[1], Gabriel T. Mindler[1], Andreas Kranzl[2], Julia Hahne[3], Rudolf Ganger[1]

[1]Department of Pediatric Orthopaedics, Orthopaedic Hospital Speising, Vienna, Austria; [2]Laboratory for Gait and Movement Analysis, Orthopaedic Hospital Speising, Vienna, Austria; [3]Orthopaedic Hospital Speising, Vienna, Austria

**LEVEL 1/Clubfoot**

Keywords: Clubfoot, Ponseti method, Gait analysis, Oxford foot model, Radiographs

*Purpose*: There is no consensus on the value of radiographs and their correlation with function or pain in clubfoot treated with the Ponseti method. We aimed to evaluate whether radiographic measurements correlate with gait parameters from the Oxford foot model and/or with a functional scoring system.
*Methods*: We included consecutive patients from our prospective database of clubfoot treated with the Ponseti method. Patients were treated within the first 3 months of life, with a minimum age of 3 years at follow up. Patients who had open joint surgery, positional clubfoot, neurological or syndrome associated clubfoot were excluded. One hundred twenty-five patients with 199 clubfeet met the inclusion criteria and 36 patients (29 %) presented for gait analysis and gave consent for radiographs. A 3D gait analysis was performed using the Oxford foot model. Radiographic angles of antero-posterior (AP) radiographs and lateral radiographs in maximum dorsiflexion and in standing position were measured. The disease specific instrument score (DSI score) was obtained using a questionnaire. Pearson correlation was calculated and tested for significance with a $p$ value below 0.05 considered as significant.
*Results*: The full set of gait data was available for 31 of 36 patients with 49 clubfeet (18 bilateral, 13 unilateral) with a mean age of 5.9 years (range 3.1–8.3). The DSI score was available for 29 of the 31 patients. A significant correlation was found between the calcaneus-first metatarsal angle in lateral maximum dorsiflexion and the standing view and parameters characterizing forefoot hindfoot sagittal motion. Further significant correlations were seen between the lateral talo-calcaneal angle in maximum dorsiflexion and hindfoot motion in relation to the tibia. The antero-posterior talocalcaneal angle showed significant correlations with parameters characterizing forefoot supination in relation to the hindfoot. The antero-posterior talus-first metatarsal and calcaneus-fifth metatarsal angle correlated significantly with forefoot–hindfoot motion in the transverse plane describing the adduction motion of the foot. The talo-calcaneal angle on the antero-posterior view and the tibio-calcaneal and the tibio-talar angle in the lateral maximum dorsiflexion view showed a significant correlation with the DSI. No significant correlation with the radiographic measurements and gait data was found when analyzing only the DSI questions related to pain.
*Conclusions*: Our results show a good correlation of measurements from radiographs and certain gait parameters. It seems that the Oxford foot model is sensitive and reflects even subtle changes between forefoot-hindfoot and hindfoot-tibial motion. Correlation of the DSI and radiographic measurements were found but must be viewed with caution due to a ceiling effect in the DSI variable. However, neither gait analysis nor radiographic measurements showed a significant correlation with pain, which is arguably the most important outcome parameter.
*Significance*: Certain radiographic measurements correlate well with foot motion in the Oxford foot model. Neither radiographs nor gait parameters seem to have a correlation with pain.

**April 3rd**
DDH/Legg-Calvé-Perthes
14:15–15:30
CONCERT HALL
**OP20**/14:15–14:25

## Expectations of patients and their surgeons of outcomes after PAO surgery

**Gloria N. Boye**, Patricia Miller, Young-Jo Kim, Michael B. Millis

Boston Children's Hospital, Boston, United States

**LEVEL 2/DDH**

Keywords: DDH, PAO, Patient satisfaction

*Purpose*: The fulfillment of patients' expectations is an essential factor in their satisfaction with outcomes of their surgery. Therefore, it is necessary to assess whether patients have realistic expectations based on adequate understanding of the nature of their symptoms and the likely effects of the prospective surgery on their condition. Comparison of the clinical outcomes anticipated by the patients and their surgeons provides an effective means of making this assessment. The expectations and satisfaction of hip impingement patients who underwent preservation surgery have been described. Such a study on expectations and satisfaction with outcomes has not yet been reported on patients undergoing periacetabular osteotomy (PAO) to treat Developmental Dysplasia of the Hip (DDH). This study examined the level of agreement in the preoperative expectations of patients with DDH and their surgeons of the PAO surgery.
*Methods*: Patients and surgeons independently completed pre-operative questionnaires on their realistic expectations of improvement in six domains representing different hip symptoms after surgery. Domains included pain, stiffness, locking, stability, walking ability and athletic ability. Two surgeons and their combined 50 patients (82 % female; mean age of 26.8 ± 9.7 years) participated in the study. Questionnaire responses regarding expectation were rated on a scale of 1 to 4 from 'not improved at all' to 'greatly improved'. Concordance between patient and surgeon expectation was evaluated by the percent of exact and partial (within one rating) agreement as well as Kappa coefficients.
*Results*: Exact agreement between patients and surgeons ranged from 15 to 51 %. Agreement was lowest regarding expected stiffness of the hip and highest regarding expected pain following surgery. Partial agreement between patients and surgeons ranged from 48 to 100 %, where the lowest partial agreement was with respect to stiffness of the hip and the highest partial agreement percentages were with respect to pain (100 %), stability of the hip (77 %) and athletic ability (70 %). Weighted Kappa estimates were low ranging from 0.08 to 0.37. In instances of disagreement, patients consistently had higher expectations than the surgeon especially with respect to athletic ability, walking ability and stiffness of the hip. Patient expectations were not found to correlate with the preoperative functional status of their hips. Pain was the only symptomatic domain reported by every patient.
*Conclusions*: The expectations of patient and surgeon were rarely in exact agreement, though the discrepancy was not very wide in most domains. The surgeon and patient most commonly anticipated similar outcomes with regards to hip pain. Our findings highlight the need for clearer understanding between patients and surgeons on the effectiveness of the PAO in improving hip stiffness, walking and athletic ability. Discrepancies in patient-surgeon expectations may predispose to poor satisfaction with treatment outcomes after PAO surgery.
*Significance*: A study on expectations and satisfaction with outcomes has not yet been reported on patients undergoing periacetabular osteotomy (PAO) to treat the residue of Developmental Dysplasia of the Hip (DDH).

**OP21**/14:25–14:35

### Hip dysplasia in the contralateral hip in unilateral late-detected hip dislocation—50 years follow-up of 48 patients

**Terje Terjesen**

Orthopaedic Department, Oslo, Norway

**LEVEL 4/DDH**
Keywords: Developmental dislocation of the hip, Contralateral hip, 50 years follow-up

*Purpose*: The aim of this study was to find the incidence of dysplasia in the "normal" contralateral hip in unilateral developmental dislocation of the hip (DDH) and to evaluate the long-term prognosis of such hips.
*Methods*: 48 patients (40 girls and 8 boys) were treated for late-detected unilateral DDH during the period 1958 to 1962. After preliminary skin traction, closed reduction was achieved at a mean age of 17.8 months in all except one patient who needed open reduction. The contralateral hip had undergone early derotation femoral osteotomy (within 3 years after reduction) in 25 patients and later surgery in nine patients. Radiographs during childhood and adulthood were reviewed. The latest radiographic follow-up was at a mean patient age of 50.9 years (43–55).
*Results*: Eight patients (17 %) developed hip dysplasia, defined as CE-angle <20° in the contralateral "normal" hip. Five of these patients underwent surgery during childhood or adolescence to improve femoral head coverage. The dysplasia improved in two patients after varus femoral osteotomy and in two patients after acetabular shelf operation. The dysplasia deteriorated to subluxation in two patients (CE-angles 4° and 5°) who both developed osteoarthritis during long-term follow-up, and one of these patients underwent total hip replacement at an age of 49 years.
*Conclusions*: Although hip dysplasia developed in 17 % of the contralateral hips, the long-term prognosis was relatively good since osteoarthritis occurred in only two hips (4 %) after a mean follow-up of 50 years.
*Significance*: Regular follow-up of the "normal" side is indicated and surgical correction in cases of subluxation is recommended.

**OP22**/14:35–14:45

### Risk factors for developmental dysplasia of the hip: a population-based cohort study

**Andreas Roposch**, Evangelia Protopapa, Saboura Mahdavi, Yael Gelfer, Olivia Malaga-Shaw, Ajay Asokan, Paul Humphries, Rumana Omar

Great Ormond Street Hospital for Children; UCL Institute of Child Health, London, UK

**LEVEL 1/DDH**
Keywords: DDH, Risk factors, Prospective cohort study

*Purpose*: To determine risk factors for DDH using newly developed diagnostic criteria based on international consensus.

*Methods*: In this population-based cohort study, 13,278 babies born at a secondary care center (2010–2013) received a standardized examination within 24 h post-partum, in which we prospectively ascertained the presence of the common risk factors for DDH (breech, family history, etc.). Infants exhibiting = 1 factor were eligible and underwent ultrasound done at 8 weeks. Alpha angles were measured by surgeon/radiologist in consensus and blinded to risk factors and age. We analyzed the association of the risk factors and ultrasonographic DDH using criteria based on international consensus, which define alpha <55° at 8 weeks as DDH.
*Results*: Of 2,276 (17 %) eligible newborns, 2,191 (96 %) were included in the study. 2,101 (92 %) infants completed follow-up with DDH present in 76 hips (3.6 %). Two strong independent risk factors were identified using multivariate analysis (odds ratio): foot deformity warranting physiotherapy or orthopaedic followup (4.52, $p = 0.019$), family history of DDH (4.42, $p < 0.001$). Of 139 infants who had one or both of these risk factors, 45 (32 %) had DDH. An abnormal hip examination was indicative of DDH (56.24, $p < 0.001$).
*Conclusions*: In this prospective study, a foot deformity was associated with a four-fold increased risk for DDH as was a family history of DDH.
*Significance*: Newborns with a foot deformity warranting physiotherapy or orthopedic followup had an increased risk for DDH.

**OP23**/14:45–14:55

### Avascular necrosis of the femoral head after failed Pavlik Harness treatment in Developmental Dysplasia of the Hip

**Madhu Tiruveedhula**, Nicholas Clarke

Southampton General Hospital, Southampton, UK

**LEVEL 3/DDH**
Keywords: Failed Pavlik harness, Developmental dysplasia of hip, Avascular necrosis of femoral head

*Purpose*: Avascular necrosis (AVN) of the femoral head is an irreversible complication seen in the treatment of Developmental Dysplasia of Hip (DDH) with Pavlik Harness. Its incidence is reported to be low after successful reduction of hip and high if the hip is not relocated. We aim to investigate its incidence after failed Pavlik harness treatment.
*Methods*: We prospectively followed a group of children who failed Pavlik harness treatment for DDH treated at our institution by senior author between 1987 and 2001 and compared their rates of AVN with a group of children who presented late and hence were not treated with Pavlik harness. AVN was graded as described by Kalamchi and MacEwen and only Grade 2–4 AVN was considered significant and included in the analysis.
*Results*: Thirty-seven hips were included in the Failed Pavlik Group (group 1) and 86 hips in no Pavlik Group (Group 2). Ten hips in Group 1 developed AVN (27 %) while only 7 hips in Group 2 (8 %) developed AVN. The odds of developing AVN after failed Pavlik treatment was 4.7 (95 % confidence interval 1.3–14.1) ($p = 0.009$) with relative risk of 3.32 (1.37–8.05). There was no statistically significant association noted with duration of harness and severity of AVN (Spearman's correlation −0.46, $p = 0.18$), and age at presentation.
*Conclusions*: We therefore advise close monitoring of hips in a Pavlik harness and discontinue its use if the hips are not reduced within 2 weeks in harness.
*Significance*: Due to the high risk of AVN, treatment with Pavlik Harness should be used cautiously in children with a fixed dislocation of hip.

**OP24**/14:55–15:05

### Extent of physeal involvement in Legg-Calve-Perthes disease

**Kwang-Won Park**, Hae-Ryong Song

Korea University Rare Diseases Institute, Seoul, Korea

**LEVEL 2/Legg-Calve Perthes disease**
Keywords: Legg-Calvés-Perthes disease, Physis, Premature closure

*Purpose*: The extent of growth plate involvement in Legg-Calvé-Perthes disease (LCPD) appears to be associated with regeneration of the affected femoral head and clinical outcomes. The growth plate involvement (GPI) index is reportedly a reliable predictor of final radiographic outcome in LCPD.

*Methods*: We retrospectively reviewed the serial radiographs of 47 skeletally mature patients with unilateral LCPD who were treated conservatively. The mean duration of follow-up was 8.9 years (range 4–13 years). The affected hips were categorized into those with and without physeal involvement. Moreover, the GPI indices were estimated and Herring classifications were determined at the initial stage of the disease. The Stulberg classification, leg length discrepancy (LLD), articulo-trochanteric distance (ATD index), neck-shaft angle, neck width and height were determined at skeletal maturity.
*Results*: The GPI indices were lower in Herring groups A and B ($p < 0.001$) and Stulberg classes I and II ($p = 0.002$), and these values were increased in the Herring Groups B/C and C and Stulberg classes III, IV and V. However, the age of onset, LLD and ATD index at skeletal maturity were not associated with the GPI index ($p = 0.226$ and $p = 0.065$, respectively).The neck-shaft angle of the affected hips with physeal involvement was significantly different compared to that of unaffected hips ($p < 0.001$).
*Conclusions*: The GPI index can predict the final radiographic outcome in LCPD patients who were treated conservatively. However, GPI index cannot predict the final amount of LLD and trochanteric overgrowth. Moreover, a younger age of onset may not result in a lesser degree of physeal involvement.
*Significance*: We can predict the final radiographic outcome in LCPD patients who were treated conservatively using GPI index, and also can decide proper treatment modality for functional deficit related to the anatomical changes.

**OP25**/15:05–15:15

## Triple osteotomy of the pelvis for Legg- Calve-Perthes disease: long term results

**R. Pailhé**, E. Cavaignac, J. Sales De Gauzy, F. Accadbled

Hôpital des Enfants, Toulouse, France

**LEVEL 3/Legg-Calve Perthes disease**
Keywords: Perthes, Triple osteotomy of the pelvis, Long term outcome, Hip

*Purpose*: Some patients with Legg-Calvé-Perthes disease (LCPD) present in their second and third decades of life with substantial dysfunction and pain. Triple osteotomy of the pelvis (TOP) has been proven to provide satisfactory short and midterm results. We aimed at evaluating those results in the long term.
*Methods*: This is a prospectively accrued series of TOP in LCPD patients operated between 1989 and 2005 at a single institution. Indication for surgery included “head at risk” signs, late onset, and Catterall classification. After 1992, Herring type was considered. Only grades B and C were eligible for surgical treatment. The end point of the study was reoperation for any cause related to the hip. Clinical evaluation was assessed by Oxford Hip Score (OHS). Radiological evaluation included measurement of the following angles: lateral center edge angle, anterior centre-edge angle, Green index, acetabular index and neck shaft angle. Stulberg and Tönnis classifications were applied. Adequate statistical tests were carried out. Factors affecting survival and functional outcome were sought.
*Results*: Forty five patients were included and reviewed at a mean follow up of 15.2 years (range 8.0–24.0). 2 patients were lost to follow up. There were 32 males and 11 females. Mean age at diagnosis was 6.1 years (range 3.0–12.0). Mean duration of symptoms before surgery was 6.6 months (range 1.0–24.0). LCPD was bilateral in one case. Two patients underwent revision a mean 7 years after the index procedure. The first case was a male patient with a leg length discrepancy of 2.5 cm. Contralateral distal femoral ephysiodesis was performed. The second revision was a male patient with retroverted femoral neck. The patient was treated with femoral osteotomy, then required 4 years later a total hip replacement. Cumulative survival rate for all TOP was 86.8 % (95 % CI 85.3–94.6 %). Mean OHS was $14.03 \pm 6.13$ (range 12–40). Age at diagnosis was associated with a 2.28 increased risk of OHS score >25 for each increased year of age ($p = 0.035$). A Cox-proportional hazard model demonstrated a significantly increased risk of revision with Green index (0.147 for each increased percentage of Green index, $p = 0.043$). Only two patients (excluding the patient who had a total hip replacement) presented with bad functional results with OHS >25. There was no significant change in radiological parameters from postoperative to final follow up. Only 2 patients presented osteoarthritis: the one who required a total hip arthroplasty was Tönnis 3, the second was asymptomatic and rated Tönnis 2. Six patients (14 %) were Stulberg I, 19 (44 %) Stulberg II, 11 (25.5 %) Stulberg III, 6 (14 %) Stulberg IV and 1 (2.5 %) Stulberg V.

*Conclusions*: Few studies have reported on the outcome of TOP in LCPD with long term follow-up. Ours demonstrated good long term survival. Considering risk factors, only Green index for survival and age at diagnosis for functional outcome were significant. Pincer-type femoroacetabular impingement and acetabular retroversion have been reported. In our study, one patient presented clinical signs of impingement with cross-over sign indicating a retroverted acetabulum. It is our belief that "overdoing" the containment is responsible for this, and thus it should be avoided.

*Significance*: TOP for LCPD can provide satisfactory and reproducible long-term clinical and radiological results in selected patients, without the limitations of femoral varus osteotomy (limp, limb shortening) and Salter osteotomy (incomplete containment).

**OP26**/15:15–15:25

### Prognostic factors for femoral head deformity in the early stages of Legg-Calve-Perthes disease: MRI diffusion and perfusion indices

Won Joon Yoo[1], **In Ho Choi**[1], Tae-Joon Cho[1], Eun Seok Choi[1], Chin Youb Chung[2], Moon Seok Park[2], Jung-Eun Cheon[1]

[1]Seoul National University Children's Hospital, Seoul, Korea; [2]Seoul National University Bundang Hospital, Seongnam, Korea

**LEVEL 2/Legg-Calve Perthes disease**

Keywords: Diffusion MRI, Perfusion MRI, Prognosticator, LCP

*Purpose*: To find prognostic factors for later femoral head deformity using the diffusion and perfusion MRI performed in the early stages before collapse of the femoral head.

*Methods*: Diffusion and perfusion MRI was performed in 46 patients (M:F = 37:9) with unilateral Legg-Calvé-Perthes disease in the stages of increased density (n = 38) and early fragmentation (n = 8). The average age was 7.5 years (range 3.3–11.9). The Perfusion Coefficient was measured in the femoral epiphysis (total epiphysis, lateral third, central third, and medial third) and the metaphysis of the proximal femur. Apparent diffusion coefficient (ADC) was measured in the femoral epiphysis and the metaphysis of the proximal femur. Perfusion difference ratio and ADC difference ratio were defined as a percentage increase or decrease compared to the normal side. The presence or absence of physeal abnormalities and metaphyseal cysts were investigated in the conventional MRI. Deformity index was calculated on plain radiographs taken at 1.9 years after symptom onset (range 1.2–2.8). Correlations between the deformity index set at 0.3 and the significant MR indices determined from the univariate analysis (correlation coefficients) were investigated using the multiple logistic regression test. Statistical significance was set at $p = 0.05$.

*Results*: Significant variables based on the univariate analysis were ADC difference ratio in the metaphysis, perfusion difference ratio in the central epiphysis, Perfusion Difference ratio in the metaphysis, physeal abnormalities and metaphyseal cyst. However, multiple logistic regression analysis revealed that only ADC difference ratio in the metaphysis ($p = 0.003$) and Perfusion Difference ratio in the central epiphysis ($p = 0.034$) were significantly associated with the deformity index.

*Conclusions*: Increased diffusion in the metaphysis and decreased perfusion in the central epiphysis in the early stages before collapse of the femoral head were associated with the deformity index at 1.9 years after symptom onset.

*Significance*: Perfusion and diffusion MRI performed at the early stages of Legg-Calvé-Perthes disease before collapse of the femoral head provides prognostic information for later deformity.

**April 3rd**
Hip/Lower Extremity/Foot and Ankle
16:00–17:30
CONCERT HALL
**OP27**/16:00–16:10

### Biomechanical comparison of semi-rigid paediatric locking nail versus titanium elastic nails in a femoral fracture model

**Marianne Flinck**[1], Johan Von Heideken[2], Per-Mats Janarv[2], Veronica Watz[3], Jacques Riad[1]

[1]Department of Orthopaedics, Skaraborg Hospital, Skovde, Sweden; [2]Department of Women's and Children's Health, Karolinska Institutet, Stockholm, Sweden; [3]Department of Solid Mechanics, Royal Institute of Technology, Stockholm, Sweden

**LEVEL 2/Hip/Lower extremity**
Keywords: Femoral shaft fracture, Children, Flexible intramedullary nail, Biomechanics, Fracture fixation, End caps

*Purpose*: The purpose of this biomechanical study was to assess the stability of a length-unstable oblique midshaft fracture in a synthetic femur model stabilized with different combinations of intramedullary elastic nails and with a pediatric locking nail.
*Methods*: Twenty-four femur models with an intramedullary canal diameter of 12 mm were used. Three groups with different combinations of titanium elastic nails with end caps (TEN) and one group with a pediatric locking nail (PLN) were tested. An oblique midshaft fracture was created, and the models underwent compression, rotation, flexion/extension, and a varus/valgus test, with 50 and 100 % of the forces generated during walking in corresponding planes.
*Results*: We present the results from 100 % of loading during walking (median and range). In axial compression, the PLN was less compressed than the combination of two 4.0 mm TEN (4.4 mm (3.4–5.4) respectively 5.2 mm (4.8–6.6); $p = 0.030$). No difference was found in compression between the PLN and the four 3.0 mm TEN (7.0 mm (3.3–8.4); $p = 0.065$). The two 3.0 mm TEN did not withstand the maximum compression of 10.0 mm. In external rotation, the PLN rotated 12.0° (7.0–16.4) while the TEN models displaced more than the maximum of 20.0°. No model withstood maximal rotation of 20.0° internal rotation. In the four-point bending test, in the coronal and the sagittal plane, all combinations except two 3.0 mm TEN in extension, withstood the maximum angulation of 20.0°.
*Conclusions*: We conclude that the PLN provided the greatest stability in all planes compared to the TEN models with end caps, even if the difference from the two 4.0 mm TEN model was small.
*Significance*: This study can guide the selection of intramedullary elastic nails to use for optimal stability. We speculate that the PLN with a greater stability could provide a faster and less painful rehabilitation in femur fractures and, in addition, provide increased stability when performing derotation osteotomies.

**OP28**/16:10–16:20

### The evolution of lower extremity deformity management In MPS IVA, Morquio–Brailsford syndrome

**Tom Southorn**[1], George Cooper[2], Deborah Eastwood[1], Ed Bache[2]

[1]Great Ormond Street Hospital, London, UK;
[2]Birmingham Children's Hospital, Birmingham, UK

**LEVEL 4/Hip/Lower extremity**
Keywords: Morquio, Mucopolysaccharidosis, Valgus

*Purpose*: Surgical interventions in children with Morquio–Brailsford syndrome type A (MPS-IVA) were historically performed only for symptom relief. With advances in management these children are surviving longer and functioning at a higher level. Surgical intervention in the lower extremity is now aimed at maintaining alignment and mobility. The purpose of this study is to describe the evolution of surgical intervention for deformity management of the lower extremity with particular reference to genu valgum and guided growth.
*Methods*: A retrospective review of notes and radiographs from two institutions of MPS-IVA patients who underwent surgery for lower extremity deformity correction was undertaken. Demographic details, surgical details, clinical measurements and outcomes, and complications were recorded as well as data for mobility pre- and post-intervention.
*Results*: 46 patients with MPS-IVA were identified. Of these, 24 patients had 8 plates and 2 had staples inserted for correction of genu valgum. 2 patients had surgery for hip subluxation. The male to female ratio was 1:1. The indication for surgery in all patients was progressive genu valgum. The average age at insertion was 8 years 6 months (4 years 7 months–15 years 11 months). Average duration of insertion was 22 months (7–60 months). Mean intermalleolar distance pre-operatively was 12.5 cm (5.3–24) and post operatively was 7.8 cm (0–23). Alignment was improved in all patients when inserted at younger than 7 years of age. Removal of plates in patients under the age of 6 was associated with higher risk and severity of recurrence. 3 patients have had 8 plates re-inserted for recurrence but with no success in further reduction of deformity. 20 patients had hip radiographs analysed. All hips were radiographically abnormal, with 17 out of 26 hips dislocated. The average Migration Percentage was 67 %, with only one hip centred in the acetabulum. Of the 2 hips treated surgically, neither had remained reduced at most recent follow up.

*Conclusions*: Progressive deformity is a well-recognised feature of MPS-IVA. Early intervention with guided growth is effective at correcting deformity but care needs to be taken to watch for recurrent deformity over time. Due to early closure of physes associated with MPS-IVA we would suggest intervention under the age of 7 years for progressive genu valgum but plates may need to be revised/retained until after closure of the physes to prevent recurrence of deformity. Increasing use of enzyme replacement therapy (ERT) particularly in children under the age of 4 may lead to further changes in the surgical management of this condition.

*Significance*: Knowledge from studies looking at the use of 8 plates in a normal physis may not be applicable to the pathological physis. In particular, the deformity in patients with MPS IV-A may need to be addressed at an earlier age and metalwork left in for longer than previously expected.

**OP29**/16:20–16:30

### The diagnostic value of a Spect-Bone scan in the assessment of femoral epiphyseal vascularity after surgical dislocation for slipped upper femoral epiphysis

**Kyle James**[1], Oliver Birke[2], Paul Gibbons[2], Manoj Ramachandran[1], David Little[2]

[1]The Royal London and Barts and The London Children's Hospital, London, UK; [2]The Children's Hospital at Westmead, Sydney, Australia

**LEVEL 2/Hip/Lower extremity**

Keywords: Slipped epiphysis, Femur, SCFE, SUFE, Bone scan, SPECT, Sensitivity and specificity, Predictive value of tests, Technetium Tc 99, Radionuclide imaging, Ischemia/etiology, Postoperative period

*Purpose*: The evaluation of intra-operative monitoring and early post-operative bone scan with Single Photon Emission Computed Tomography (SPECT) in assessing the vascularity of the femoral head after the treatment of slipped capital femoral epiphysis (SCFE) with a surgical hip dislocation approach. Secondarily, to correlate the vascularity of femoral head using intra-operative monitoring and/or SPECT-Bone scan with radiological evidence of femoral head collapse at a mean follow-up period of 1.3 years.

*Methods*: At our institution, from April 2011, intra-operative monitoring of the femoral head perfusion was performed using an ICP Pressure Probe (Codman®). All patients had a post-operative 3 phase bone scan performed at day 5–7 with delayed phase pin-hole, SPECT and CT images that were reported by a Nuclear Medicine Radiologist.

A vascularised femoral head on SPECT Bone scan was classified as viable. Patients then commenced weight bearing at 6 weeks. A non-vascularised femoral head was classified as non-viable and patients were managed with 12 months non-weight-bearing (NWB) and bisphosphonate treatment.

*Results*: Of the 77 SCFEs treated since April 2011, 38 slips in 37 patients (18 female:19 male) had sufficient follow-up for analysis (mean 1.3 years; 0.9–2.3 years). There were 22 stable (1 bilateral) and 16 unstable SCFEs. The mean posterior slip angle (PSA) was 58 (40–85) in stable slips and 62 (35–82) in unstable slips.

In the stable group 21/22 were pulsatile on intra-operative monitoring; all 22 were viable on SPECT-bone scan. One case (chronic, severe slip PSA 85) with no intra-op pulse later collapsed. Stress shielding osteopenia of the femoral head in this case may have resulted in collapse.

In the unstable group 12/16 were pulsatile on intra-operative monitoring, with 10/16 viable on SPECT. There was no collapse of SPECT viable cases. Of the 6 non-viable cases 3 showed varying degree of collapse. Defining the non-viable SPECT cases as the 'at risk group' for collapse. Both intra-op pulsatile cases with non-viable SPECT collapsed to some degree.

Pulsatile intra-operative monitoring alone had a positive predictive value (PPV) of 100 % for no collapse in stable and 83 % in unstable slips, a negative predictive value (NPV) of 100 % in stable and 25 % in unstable SCFE.

A viable SPECT-bone scan had a 95 % PPV for no collapse in stable SCFE (0 % NPV), and 100 % PPV and 50 % NPV in unstable SCFE.

Pulsatile intra-operative monitoring combined with a viable SPECT-bone scan had a 100 % PPV for no collapse in stable and unstable SCFE, a 100 % NPV in stable and 50 % in unstable SCFE.

*Conclusions*: In stable SCFE the intra-operative monitoring showed a 100 % positive predictive value of no collapse and, once established, may be sufficient alone to assess femoral head vascularity—complemented by post-operative SPECT-bone scan if not conclusive intra-operatively.

In unstable SCFE a viable post-operative SPECT-bone scan was 100 % predictive of no collapse and therefore complements the predictive value of the intra-operative monitoring.

A non-viable SPECT-bonescan had a 50 % NPV of collapse under the treatment regime of NWB and bisphosphonates at 1 year.
*Significance*: Intra-operative monitoring and SPECT-bone scan are both valuable in assessing the vascularity of the femoral head following surgical dislocation in SCFE and can be useful in guiding post-operative return to weight bearing and treatment in an aim to prevent collapse of the non-viable 'at risk' cases.

**OP30**/16:30–16:40

### Stature lengthening using the Precice implantable lengthening nail

Kevin Debiparshad, David Chong, **Dror Paley**

Paley Advanced Limb Lengthening Institute, West Palm Beach, USA

**LEVEL 3/Hip/Lower extremity**
Keywords: Stature, Precice nail, Limb lengthening

*Purpose*: Stature lengthening is performed for short stature for dysplasias and cosmetic (height dysphoria) indications. With the advent of better implantable lengthening devices this technique has become more practical and appealing. This study was performed to evaluate the use of the PRECICE™ (Ellipse Technologies, Irvine, CA, USA), a recently FDA approved lengthening nail for bilateral femoral and tibial lengthening for short stature.
*Methods*: We retrospectively reviewed 26 stature lengthening patients; 24 cosmetic and 2 achondroplastic. Three cosmetic and both achondroplastic patients had sequential or simultaneous femur and tibia lengthening. Two cosmetics had only tibial lengthening and 19 only femoral lengthening. Both achondroplastics and 4 cosmetics were female and 20 cosmetics male. The achondroplastics were 14 and 16 years old. The cosmetics were: mean 28 years (15–51).
*Results*: Mean lengthening per segment was 5.2 cm (3–6.5). Mean height gain was 6.1 cm (3–11.8). There was one femur fracture and 3 femoral nails that fractured at the weld, that required nail replacement. The distraction mechanism broke in three nails, limiting further lengthening. There were 2 ilio-tibial band contractures requiring release, one premature consolidation treated by reosteotomy, two delayed ossification, and 1 DVT. There was one achondroplastic deep femoral infection treated by an antibiotic cement rod. In total there were 10 unplanned surgical procedures. All complications were successfully treated with no loss of length.
*Conclusions*: This preliminary study demonstrates the reliability and complications of the PRECICE™ for stature lengthening. Rate control and ability to achieve desired length was excellent. The welds in the nail and the magnet-drive shaft junction were identified as potential failure points.
*Significance*: This is the first series of stature (bilateral) lengthenings using the PRECICE™ nail. A redesign to strengthen the drive shaft junction and eliminate the welds with a one-piece casing is indicated.

**OP31**/16:40–16:50

### Demographics and management of slipped capital femoral epiphysis between 2007 and 2012: a four centre UK study

Fiona Bintcliffe[1], Antoine De Gheldere[2], Simon Thomas[1], Rachel Buckingham[3], **Manoj Ramachandran**[4]

[1]Bristol Royal Children's Hospital, Bristol, UK; [2]The Great North Children's Hospital, Newcastle upon Tyne, UK; [3]Oxford Children's Hospital, Oxford, UK; [4]The Royal London and Barts and The London Children's Hospitals, London, UK

**LEVEL 4/Hip/Lower extremity**
Keywords: Slipped capital femoral epiphysis, Demographics, Management

*Purpose*: To inform a working group of UK paediatric orthopaedic surgeons (the UK SCFE Study Group) convened to design contemporary trials in slipped capital femoral epiphysis (SCFE), four centres (Bristol, Newcastle, Oxford, and Barts and The London) reviewed the demographics and management of children with SCFE presenting to their institutions between 2007 and 2012.
*Methods*: At all contributing centres with digital radiographic (PACS) records for a minimum of 5 years, a search for the following terms was made of PACS reports: slipped capital femoral epiphysis, slipped upper femoral epiphysis, SCFE and SUFE. From the results, radiographs and electronically stored clinical records were assessed to confirm the diagnosis and determine the age at presentation, incidence of bilaterality, chronicity, stability (Loder criteria), management and complications.
*Results*: A total of 198 SCFEs presented between 2007 and 2012 to the four units. The mean age at presentation was 12.4 years (range 6–16 years). There were 99 males and 60 females. The left hip (113) was nearly twice as commonly involved compared to the right (85), with bilateral presentation in 23.8 % of patients. The most common mode of presentation was acute-on-chronic (93 acute/acute-on-chronic, 64 chronic and 41 unknown). Stable slips were over twice (2.8) as common as unstable (149 vs 49). The most common intervention was percutaneous pinning in situ. Open reduction, osteotomy and stabilisation were required in 17.2 % of cases. The most common complications were osteonecrosis (n = 17; all in unstable slips), screw migration/penetration (n = 11), leg length discrepancy (n = 10) and symptomatic secondary CAM with femoro-acetabular impingement (n = 8).
*Conclusions*: This four-centre UK study shows that SCFE continues to be a significant problem, with a third of cases presenting as unstable over a period of 6 years. The number of complications, particularly in unstable slips, is also important to note, with osteonecrosis being a significant problem.
*Significance*: This study gives insight into the demographics and contemporary management of SCFE in the UK, and acts as a guide to larger audits of current practice and informs the subject and design of potential future randomised clinical trials.

**OP32**/16:50–17:00

**Hip dysplasia in patients with cerebral palsy treated with Bernese periacetabular osteotomy**

Mark Leland Miller, Angela Keith, John Clohisy, **Perry Schoenecker**

Washington University, St. Louis, USA

**LEVEL 4/Cerebral palsy**
Keywords: Hip dysplasia, Cerebral palsy, Periacetabular osteotomy, Neuromuscular hip dysplasia

*Purpose*: Treatment of symptomatic hip dysplasia in patients with spastic cerebral palsy (CP) is challenging and surgical options are controversial. This study examines our experience with Bernese periacetabular osteotomy, in combination with adjunctive treatments, for the treatment of hip dysplasia in patients with CP.
*Methods*: Between 2001 and 2012, 14 patients (16 hips) with symptomatic hip dysplasia associated with cerebral palsy were comprehensively treated with a periacetabular osteotomy and adjunctive procedures by the senior authors and retrospectively reviewed. The median age at surgery was 17 (range 13–30). 15 of 16 patients had Gross Motor Function Classification System (GMFCS) levels of 1 or 2 prior to surgical intervention. One patient was GMFCS level 4 preoperatively. We compared preoperative pain, validated hip functional scores and radiographic parameters of hip dysplasia to postoperative results. 14 of 16 patients had at least 12 months of follow-up with a median of 35 months of follow-up. Adjunctive treatments were performed in 10 hips: 6 hips had open adductor tenotomies and 5 hips had concomitant proximal femoral osteotomy.
*Results*: The primary outcome was the Modified Harris Hip Score (MHHS). Preoperatively, the patients had an average MHHS of 57. Postoperatively, at the most recent clinic visit (range 13–125 months), the average MMHS was 75 for an increase of 18 points (p = 0.01). The pain component of the MHHS improved from 20 to 37.
*Conclusions*: In combination with appropriate adjunctive treatments, Bernese Periacetabular osteotomy can be an effective treatment for painful hip dysplasia in patients with spastic cerebral palsy through appropriate lateral and posterior coverage of the acetabulum and limitation of migration of the femoral head. A clinically significant improvement in pain and function has been documented in long-term follow-up.
*Significance*: This is the first comprehensive study to show that Bernese periacetabular osteotomy can effectively correct acetabular deformity and hip joint subluxation associated with spastic cerebral palsy. For this selected group of patients, pain has resolved and function has notably improved. Periacetabular osteotomy should be considered as a surgical option for hip dysplasia in appropriately selected patients with spastic cerebral palsy.

**OP33**/17:00–17:10

**Detailed dynamics of the pediatric flatfoot**

**Elke Viehweger**, Guillaume Authier, Claude Pothrat, Elisabeth Castanier, Guillaume Rao, Jean-Luc Jouve

Timone Children's Hospital, Pediatric Orthopaedics, Motion Analysis Center, Aix-Marseille University, Marseille, France

**LEVEL 3/Foot and ankle**
Keywords: Flatfoot, Gait, Oxford foot model, Children

*Purpose*: In young children flexible flat foot (FF) has a prevalence of about 40 %. Usually the medial longitudinal arch elevates spontaneously. Some children complain about pain. No evidence has previously been reported that an arch could be artificially created by an external orthotic device. The incidence of FF on 3D-gait parameters is poorly described. No consensus has emerged, especially no study has sought to analyse all the aspects of motion simultaneously.

The purpose of the study was to describe biomechanical consequences of architectural anomalies in FF and to establish multifactorial discriminative criteriae including 3D-gait variables.

*Methods*: A prospective study was conducted comparing a pediatric symptomatic idiopathic FF group (10 children, 8.2 ± 3.4 years old) and a healthy children group (HF) (10 children, 8.1 ± 1.6 years old), merging standardized clinical lower limb measurements, dynamic EMG (ZeroWire AURION 1,000 Hz) 3D-gait analysis (Plug-In Gait and Oxford Foot Model) and kinetics. Statistical analyses were carried out using one-way ANOVAs ($p < 0.01$).
*Results*: FF have significantly: a larger forefoot supination, relative to the hind foot (14.2 ± 7.38 versus 1.35 ± 2.48); hamstring, adductor and gastrocnemius tightness and differences in gait variables. FF children walked with significantly externally rotated feet (15.8 ± 9.8 versus 9.7 ± 4.58). To compensate during gait, supination decreased relative to the tibia. Hip extension and ankle dorsal flexion moments were decreased. An EMG showed decreased activity of the tibialis anterior at initial contact and increased activity of soleus.
*Conclusions*: Forefoot supination is the key element of the so called pediatric idiopathic flatfoot. During weight loading that leads to excessive hindfoot valgus to reduce the constitutive deformity, including muscular compensations. Those deviations would create a strain overload in the talo-navicular joint area that would instigate the collapse of the mid-foot over time or in the presence of added pathology. This hypothesis could be tested using a biomechanical model of the child flat foot. Our results showed that the choice of a combined and multifactorial analysis of all the gait parameters is fully justified, as several elements could thus be linked.
*Significance*: Relevant criteria of FF compared to non deformed feet were determined and could now be used in daily clinical practice. Reference data are established to evaluate the outcome of orthopaedic and surgical treatment, and may be used to analyse more complex foot deformities.

**OP34**/17:10–17:20

### Gait analysis in flexible flatfoot

**Tamas Terebessy**, Laszlo Kiss, Gabor Grosz, Gyula Domos, Nikoletta Horvath, Sandor Kiss, George Szoke

Semmelweis University Department of Orthopedics, Budapest, Hungary

**LEVEL 3/Foot and ankle**
Keywords: Flatfoot kinematics dynamic

*Purpose*: Flexible flatfoot is one of the most frequent disorders in childhood. Increased femoral neck anteversion, general ligament laxity, obesity, valgus knee deformity, shortened Achilles tendon and shortened peroneal tendon are the most commonly mentioned factors in aetiology. Several clinical tests can be assessed for evaluating calcaneal valgus position, the height of the longitudinal arch, the abduction and pronation of the forefoot usually by static, weight bearing position. However less is known about these parameters under dynamic conditions. The aim of this study was to describe foot kinematics during gait in flexible flatfoot.
*Methods*: 23 patient with flexible flatfeet were included in the study. Data of 66 healthy volunteers were used as standard control. Diagnosis of flatfoot was based on the clinical finding of a severe calcaneal valgus position. 3D gait analysis was performed (Vicon Oxford Metrics) with the Oxford Foot Model marker protocol. 3D kinematics of the hindfoot and forefoot such as the dorsal/plantar flexion, the valgus/varus position of the hindfoot, the abduction/adduction, and the pronation/supination of the forefoot were measured. The frontal plane kinetics of the hip-, the knee- and the ankle were also evaluated. Results are presented as mean ± SD.
*Results*: In terminal stance −16.18 ± 9.00° vs. 3.5 ± 2.5° of calcaneal valgus position was found compared to the healthy volunteers and decreased plantarflexion was observed in the Flatfoot patient Group (FPG) (−9.75 ± 4.9° vs. −20.00 ± 7.0°). In midstance forefoot abduction was found in the FPG (−14.78 ± 9.82° vs. 3.00 ± 6.61°) and surprisingly forefoot supination was measured (24.23 ± 17.25° vs. 3.50 ± 4°). Assessing frontal plane kinetics, external varus momentum was decreased in the knee (0.14 ± 0.04 vs. 0.60 ± 0.14 Nm/kg) and also in the ankle (−0.0026 ± 0.0049 vs. 0.17 ± 0.13 Nm/kg). Non significant kinetic changes were observed in the hip joint.
*Conclusions*: In this study the authors showed the biomechanical changes in pediatric flexible flatfoot under dynamic conditions. Observations are presented on how the flatfoot interferes with physiological gait. The Oxford Foot Model is a suitable tool for quantification of calcaneal valgus position. Interpretation of the decreased plantarflexion in terminal stance is challenging, but a mild vertical position of the talus in flatfoot, which is known from radiological studies, could be an explanation. Flatfoot is defined as a pronating deformity of the foot, therefore the supinating position of the forefoot in relation to the hindfoot observed in our measurements is very remarkable. Decreased external varus momentum of the knee and of the ankle may act as increased valgus stress of the joints which is in good accordance with the finding that flatfoot may lead to lateral pain and cartilage damage of the knee joint in adulthood.
*Significance*: Biomechanics of flatfoot is presented under dynamic conditions.

**OP35**/17:20–17:30

### Spontaneous restoration of radiographic indices in idiopathic plano-valgus feet

**Moon Seok Park**[1], Kyoung Min Lee[1], Soon-Sun Kwon[1], Seung Yeol Lee[1], In Hyeok Lee[1], Sang Hyeong Lee[2], Dae Gyu Kwon[3], Chin Youb Chung[1]

[1]Seoul National University Bundang Hospital, Seong Nam, Korea; [2]Dongguk University Ilsan Hospital, Go Yang, Korea; [3]In-Ha University Hospital, In Cheon, Korea

**LEVEL 4/Foot and ankle**

Keywords: Idiopathic planovalgus, Spontaneous restoration

*Purpose*: The prevalence of idiopathic plano-valgus decreases with age in children and adolescents. The values of previous studies, based on an average of radiographic indices of patients in each age group, could not give information about the rate of spontaneous correction and the affecting factors. Therefore, the aim of this study was to estimate the rate of spontaneous restoration of radiograph indices in idiopathic planovalgus by linear mixed model application.
*Methods*: We enrolled patients with idiopathic plano-valgus who were followed up for at least 1 year and underwent weight-bearing foot radiographs (bilateral antero-posterior and lateral view) more than two times under the age of 15 years between May 2003 and December 2012. Following a reliability test, the talo-navicular coverage angle, antero-posterior talus-first metatarsal angle, calcaneal pitch angle, and lateral talus-first metatarsal angle were measured from the radiographs taken at the periodic follow-ups. The rate of angular correction was adjusted for multiple factors by using a linear mixed model with gender and laterality as the fixed effects and age and each subject as the random effect.
*Results*: A total of 568 feet were finally included in this study, and a total of 3,284 radiographs were measured. The talonavicular coverage angle, antero-posterior talus-first metatarsal angle, and lateral talus-first metatarsal angle decreased by 1.7°/year ($p < 0.001$), 2.1°/year ($p < 0.001$), and 0.7°/year ($p = 0.022$), respectively. Spontaneous restoration of the calcaneal pitch with aging was not statistically significant.
*Conclusions*: The talonavicular coverage angle, antero-posterior talus-first metatarsal angle, and lateral talus-first metatarsal angle on antero-posterior and lateral radiographs of foot improved as patients with idiopathic plano-valgus feet grew older. Information about the rate of restoration can help physicians to predict the radiographic correction of idiopathic plano-valgus.
*Significance*: When physicians treat idiopathic planovalgus in growing children, results of this study is helpful to explain to patients and predict the radiographic restoration.

**April 4th**
M-S Infections/Trauma-Lower Extremity
08:00–09:00
CONCERT HALL
**OP36**/08:00–08:10

### A clinical algorithm or hip joint aspiration: which is better for the diagnosis of septic arthritis of the hip?

**Uri Givon**[1], Amir Herman[2], Amos Schindler[1], Boaz Liberman[2]

[1]Safra Hospital for Children, Tel Hashomer, Israel; [2]Sheba Medical Center, Tel Hashomer, Israel

**LEVEL 1/Musculoskeletal infections**

Keywords: Septic arthritis, Hip joint, Hip aspiration, Ultra sound, Clinical criteria

*Purpose*: The differential diagnosis between septic arthritis of the hip joint (SAHJ) and transient synovitis (TS) is important as the former may cause devastating complications. Clinical algorithms were reported, but their value was doubted in later studies. The aim of this study was to compare diagnostic hip joint aspiration to a well accepted clinical algorithm.
*Methods*: We reviewed 231 patients with TS and 27 patients with SAHJ who underwent a hip joint aspiration under ultra-sound (US) on admission to the hospital. The US findings—thickened joint capsule and increased joint width $\geq 5$ mm, and the aspirated fluid cell count and appearance were compared to Kocher's criteria (KC). The diagnostic value was calculated using sensitivity (Sen), specificity (Spec), positive and negative predictive values (PPV; NPV) and the number needed to diagnose (NND). P value of less than 0.05 was considered as statistically significant. All p values are two sided.
*Results*: The value of the KC was found to be ideal when we used 2 or more criterions for the diagnosis SAHJ. In this case the NND was 2.07. The value of US findings for the diagnosis of SAHJ was found to be low, i.e., PPV and NPV for thickened joint capsule were 15.3 and 85.5 %, respectively; PPV and NPV for the difference of joint width over 5 mm were 9.6 and 79.1 %, respectively. The highest diagnostic value was found for the hip joint aspiration findings. The NND was the lowest (1.03) when either the leukocyte count was higher than 50 K/dL or the aspirate was opaque. This low NND represents a very high sensitivity (100 %) and specificity (96.2 %), twice as good as the best NND for KC.
*Conclusions*: Hip joint aspiration was superior to the accepted clinical algorithm. The lowest NND was found with a combination of aspirate WBC count and opacity, and it was twice as good as the best NND for KC. Hip joint aspiration is a simple procedure, can be performed under sedation and US control and is an excellent diagnostic tool to rule out SAHJ.
*Significance*: Hip joint aspiration is an excellent modality for diagnosis of SAHJ in children. We recommend performing a hip joint aspiration in patients who are unable to bear weight and are evaluated for possible SAHJ.

**OP37**/08:10–08:20

### The use of 16s ribosomal RNA for the detection of bacteria in blood, synovial and tissue of children with osteoarticular infections

Saar Hashavya, Paul Turkeltaub, Carlos Hidalgo-Grass, Naum Simanovsky, **Ron Lamdan**

Hebrew university and Hadassah Medical Center, Jerusalem, Israel

**LEVEL 2/Musculoskeletal infections**
Keywords: Osteoarticular infections, 16S ribosomal RNA

*Purpose*: Osteoarticular infections are a common cause of orthopedic morbidity in the paediatric age group.

Although at times the diagnosis is straightforward there is no "gold standard" test to define these infections and an amalgam of clinical, radiological, microbiological and laboratory tests in collaboration usually implies the diagnosis.

Of the tests mentioned above the microbiological isolation of the culprit pathogen is of paramount importance.

The use of 16s Ribosomal RNA have been shown to be as effective as the traditional synovial fluid cultures and even superior in the detection of fastidious bacteria (e.g. Kingella kingae).

However the evaluation of 16s Ribosomal RNA in comparison to blood culture as an ancillary diagnostic test of osteoarticular infections was only anecdotally previously reported in the literature.

We conducted a prospective study comparing the specificity and sensitivity of 16s Ribosomal RNA from synovial, blood and tissue of children with osteoarticular infection to "gold standard" traditional bacterial cultures.
*Methods*: Synovial, blood and tissue specimens from children with suspected osteoarticular infection were cultured using traditional bacterial growth media and were also subject to 16s Ribosomal RNA amplification using QIAamp DNA Mini (QIAGEN) and general amplification reaction intended to amplify any Eubacteria. Results were correlated with the patient's clinical and laboratory data.
*Results*: Of the children enrolled in the study, 14 % (4/28) had positive blood cultures and 25 % (4/16) had positive synovial cultures. Overall 16s Ribosomal RNA positive blood and synovial cultures rate was 46 % (13/28). The main pathogen involved in osteoarticular infection using the 16s Ribosomal RNA was kingella kingi followed by *Staphylococcus aureus*.
*Conclusions*: 16s Ribosomal RNA PCR is an effective ancillary test in the detection of osteoarticular infections. Despite the overall low yield of blood cultures in the detection of osteoarticular infections, 16s Ribosomal RNA PCR is not inferior to the gold standard "blood cultures" and may add a timely, accurate adjunct to the diagnosis of osteoarticular infections. Our findings also support the notion that Kingella Kingi has become a major pathogen in paediatric osteoarticular infections.
*Significance*: This is the first report of the use of the 16s PCR method in blood samples and its correlation with findings in synovial fluid. With current technique the results obtained were similar to blood cultures' yield but faster, potentially allowing earlier initiation of appropriate treatment

**OP38**/08:20–08:30

### Interleukin-6 in paediatric musculoskeletal infection: a pilot study

**Byron F. Stephens**[1], Jeffrey R. Sawyer[1], William C. Warner[1], James H. Beaty[1], David D. Spence[1], Alice A. Moisan[2], Derek M. Kelly[1]

[1]University of Tennessee/Campbell Clinic Department of Orthopaedic Surgery, Memphis, United States; [2]Le Bonheur Children's Hospital, Memphis, United States

**LEVEL 2/Musculoskeletal infections**
Keywords: Interleukin-6, Infections, Musculoskeletal, Pediatric, Pilot

*Purpose*: Pediatric musculoskeletal infection represents a diagnostic challenge and the clinical consequences of delayed treatment can be devastating. While many institutions utilize the criteria identified by Kocher et al. in evaluating patients with suspected musculoskeletal infection. Others have called the validity of this model into question. In addition, interleukin-6 (IL-6) has recently been identified as the most sensitive marker of prosthetic joint infection (6). Our study seeks to evaluate the utility of IL-6 as a (1) diagnostic tool and (2) marker of therapeutic response to treatment in paediatric patients presenting with suspected musculoskeletal infection.

*Methods*: After obtaining IRB approval and informed consent, IL-6 serum levels were collected prospectively in 12 pediatric patients with suspected musculoskeletal infection. Patients with known immunodeficiency were excluded. These samples were then assayed in triplicate using a Quantikine ELISA assay from R&D Systems (Minneapolis, MN, USA). Additional data collected included demographic information, symptom duration, number of Kocher criteria, number of physicians seen prior to presentation, traditional inflammatory markers, temperature, final diagnosis, treatment procedures, and any organisms identified via intraoperative cultures.
*Results*: 8 patients were diagnosed with suppurative musculoskeletal infection. 4 patients were diagnosed with "other" aetiologies (3 with transient synovitis and 1 with superficial cellulitis). The mean IL-6 level for patients with musculoskeletal infection was 214.5 pg/ml, versus 68.63 pg/ml in patients with "other" diagnoses ($p = 0.107$). Descriptive analysis of trend line data reveals that IL-6 has the steepest slope, possibly indicating that it is a more sensitive marker of therapeutic response than the traditional markers.
*Conclusions*: In this small pilot study, we have identified preliminary evidence to support the clinical use of IL-6 in paediatric musculoskeletal infection. Continued research in this area is justified and necessary before definitive conclusions can be made regarding the clinical benefit of using this new inflammatory marker.
*Significance*: Our preliminary results justify further research efforts in this area. The use of IL-6 in the diagnosis and treatment of pediatric musculoskeletal infection could provide physicians with added confidence with both the initial diagnosis and efficacy of the treatment course.

**OP39**/08:30–08:40

### Changes in the treatment of paediatric femur fractures: trends from a United States kids' inpatient database (kid)

**Sameer Naranje**[1], Derek M. Kelly[1], Jeffrey R. Sawyer[1], Tamekia L. Jones[2], James H. Beaty[1]

[1]University of Tennessee/Campbell Clinic Department of Orthopaedic Surgery, Memphis, United States; [2]Department of Pediatrics and Preventive Medicine University of Tennessee Health Science Center/Children's Foundation Research Institute, Memphis, United States

**LEVEL 3/Trauma—Lower limb**
Keywords: Femur, Fracture, Treatment, US, Trends

*Purpose*: Pediatric femur fractures are commonly encountered by the paediatric orthopaedist, and there are many available treatment options. We are unaware of any nationwide epidemiology study from the United States reporting the trends in management of paediatric femur fractures over recent years. The purpose of this study is to report changes in the trends of treatment modalities for paediatric femur fractures in the United States from 1997 to 2009.
*Methods*: This study included estimated discharges for years 1997, 2000, 2003, 2006, and 2009 using the Kids' Inpatient Database (KID), Healthcare Cost and Utilization Project (HCUP), Agency for Healthcare Research and Quality. Data for this study were extracted using the international classification of diseases, ninth revision, and clinical modification (ICD-9-CM) for paediatric femoral fracture treatments. Patients included in this study were 0 to 17 years old and categorized into the following age groups: <1, 1 to 4, 5 to 9, 10 to 14, and 15 to 17 years. The Z-test calculator was used to test the difference in KID discharge estimates between 1997 and 2009. Statistical significance was defined as a p-value less than 0.01.
*Results*: There were a total of 64,404 estimated discharges recorded in the database for years 1997, 2000, 2003, 2006 and 2009 for treatment of pediatric femur fractures. There were fewer closed reductions of femoral fractures performed in 2009 compared to 1997 for age groups 5 to 9 years ($p < 0.0001$), 10 to 14 years ($p < 0.0001$), and 15 to 17 years ($p = 0.0001$). On the contrary, more open reductions and internal fixations of femoral fractures were performed in 2009 compared to 1997 for age group 5 to 9 years ($p < 0.0001$). Furthermore, fewer open reduction and internal fixation procedures were performed for age group 15-17 years in 2009 compared to 1997 ($p < 0.0001$).
*Conclusions*: There is an increased popularity of open reduction and internal fixation as a choice of treatment for the management of femur fractures in the 5 to 9 years age group in recent years. There is evidence that paediatric femur fractures are now treated more commonly with internal fixation, which is utilized in the younger age groups.
*Significance*: Increased awareness of changing trends in choice of treatment of paediatric femur fractures is crucial for maintaining standards of care for patients. Further studies focusing on the cause and cost effectiveness are needed to analyse the economic impact of these changing patterns of treatment for paediatric femoral fractures.

**OP40**/08:40–08:50

### All lateral versus medial and lateral flexible intramedullary nails for the treatment of paediatric femoral shaft fractures

Jason Matthew Cage[1], Sheena Rachel Black[2], Robert Lane Wimberly[3], W. Taylor Gheen[4], **Anthony Ian Riccio**[3]

[1]Tripler Army Medical Center, Honolulu, United States; [2]University of Texas Southwestern Medical Center, Dallas, United States; [3]Texas Scottish Rite Hospital for Children, Dallas, United States; [4]Children's Medical Center, Dallas, United States

**LEVEL 3/Trauma—Lower limb**

Keywords: Femur, Fracture, Flexible intramedullary nail

*Purpose*: Multiple techniques for flexible intramedullary fixation of paediatric femur fractures have been described. To our knowledge, no study comparing medial and lateral entry versus all lateral entry retrograde nails has been reported. The purpose of this study is to compare surgical and radiographic outcomes and rates of symptomatic hardware removal between these techniques.
*Methods*: An IRB approved, retrospective review of patients treated by retrograde, dual flexible intramedullary fixation of femur fractures was performed at a paediatric hospital between the years 2009-2012. Demographics, blood loss, and operative time were collected from the medical and surgical records. We assessed radiographs for fracture pattern, time to union, and canal fill as well as shortening, and angulation at the time of osseous union. Rates of symptomatic hardware and hardware removal were noted. Data was compared between patients treated with all lateral entry nailing and those treated with medial and lateral entry nailing using the Student's *t* test.
*Results*: 131 children with femoral shaft fractures were treated with retrograde flexible intramedullary fixation using Ender's stainless steel nails (Richards). 45 were treated with two lateral entry nails and 86 were treated with one medial and one lateral entry nail according to surgeon preference. There were no statistical differences in gender, age, weight, body mass index, blood loss, and fracture pattern between the two groups. The average total anesthesia time was 27 minutes faster in the lateral group ($p<0.0001$). There was no difference between the two techniques in shortening or coronal angulation at union regardless of fracture pattern. In comminuted fractures, the lateral nails demonstrated less sagittal angulation (0.4 degrees vs 3.2 degrees, $p= 0.0159$). In the lateral group, there was greater correlation between fill of the canal and reduced shortening at union. There was no statistical significance noted for shortening or angulation between entry types based upon weight. There is a trend for increased symptomatic hardware removal in the lateral group (17.5 versus 11.0 %, $p = 0.3163$) but no correlation between symptomatic hardware and BMI in either group. There were no iatrogenic neurologic injuries, infections, clinically significant malunions, non-unions, or refractures.
*Conclusions*: Medial and lateral versus all lateral retrograde flexible nails in femur fractures demonstrate comparable fracture stability and safety. The all lateral technique is potentially a faster procedure. Neither technique demonstrated a clinically significant difference in rates of shortening, angulation, or symptomatic hardware.
*Significance*: Final fracture alignment, surgical complications and rates of symptomatic hardware are clinically comparable between paediatric femur fractures treated with all lateral entry flexible nailing and those treated with medial and lateral flexible nailing.

**OP41**/08:50–09:00

### Socio-economic factors and insurance status are associated with disparities in elective paediatric orthopedic surgery

**Emily R Dodwell**[1], James Wright[2], Roger Widmann[1], Flo Edobor-Osula[1], Ting-Jung Pan[1], Stephen Lyman[1]

[1]HSS, New York, USA; [2]Hospital for Sick Children, Toronto, Canada

**LEVEL 3/Trauma—Lower limb**

Keywords: Hardware removal, Femoral shaft fracture, Health disparities

*Purpose*: Disparities in surgical outcomes and access to orthopaedic care exist in adults, and have also been identified in non orthopaedic paediatric conditions. The purpose of this study was to assess whether health disparities exist in paediatric orthopaedics, and to determine which demographic and socio-economic factors may be associated with differences in treatment.
*Methods*: Children aged 7–18 who sustained a femoral shaft fracture, and underwent femoral shaft fracture internal fixation between the years 1997 and 2010 were identified in the New York State SPARCS database. Cases in which surgical fixation was removed in the first 3 months following fixation were excluded as these were postulated to likely represented complications of surgery, and not elective hardware removal following complete healing. Patient demographics including age, sex, race, insurance status, household income, educational status and geographic location were assessed. Univariate and multivariate logistic regression analysis was performed to identify factors associated with hardware removal.
*Results*: Between 1997 and 2010, 2219 closed femoral shaft fractures were treated with internal fixation. 580 of these (26.1 %) underwent hardware removal. Younger age (<0.001), Caucasian (<0.001), higher education (0.002), and private insurance (compared to no-fault) (<0.001), and being treated by a pediatric orthopedic surgeon instead of a non pediatric specialist (0.001) were predictors of hardware removal following logistic regression analysis. There was a trend for decreased hardware removal in patients with self-pay and Medicaid compared to private insurance, but this was not statistically significant.
*Conclusions*: There is an association between decreased elective orthopaedic procedures and lower socio-economic status in paediatric orthopaedics.
*Significance*: Differences in removal of implants may represent a disparity in access to care for children in New York State. Recognition of paediatric orthopaedic health disparities may improve paediatric orthopaedic care, and have implications for fellowship training, changes to physician re-imbursement, and health care policy reform.

**April 4th**
Spine Part I
09:00–10:00
CONCERT HALL
**OP42**/09:00–09:10

## The crucial impact of T1-slope for sagittal alignment evaluation in adolescent idiopathic scoliosis

**Sebastien Pesenti**, Benjamin Blondel, Emilie Peltier, Elie Choufani, Gerard Bollini, Jean-Luc Jouve

Aix-Marseille University, Marseille, France

**LEVEL 4/Spine**
Keywords: Idiopathic scoliosis, Sagittal alignment, T1-slope

*Purpose*: Sagittal alignment has been shown to be highly correlated to outcomes scores in various spinal deformities. Recently, T1-slope defined as the angle between T1 superior endplate and the horizontal has been described and correlated with global sagittal alignment parameters. The aim of this study was to evaluate the influence of the T1-slope in the management of Adolescent Idiopathic Scoliosis (AIS).
*Methods*: 29 patients diagnosed with AIS and with a preoperative hypokyphosis (<20°) were included in this retrospective study. Surgical procedures included systematically a hybrid construct with pedicular screws below T11, sublaminar bands in the concavity of the deformity and a hook-claw at the top of the construct. Radiographic evaluation was done preoperatively and postoperatively with measurements of Cobb angle, T1-slope, T1-tilt, thoracic kyphosis, lumbar lordosis and pelvic parameters
*Results*: On the whole series, Cobb angle was significantly decreased (58° vs. 17°, $p < 0.001$), the thoracic kyphosis was significantly increased (12.6° vs. 23.7, $p < 0.001$), as well as the cervical lordosis (6° of kyphosis vs. 4° of lordosis, $p < 0.001$) and the T1-slope (9.8° vs. 16.3°, $p < 0.001$). No significant variation of the T1-tilt was noted (−1.6° vs. −1.8, $p = 0.802$), neither of the lumbar lordosis or pelvic parameters. T1-slope was significantly correlated to T1-tilt preoperatively ($r = 0.376$, $p = 0.027$). During the postoperative evaluation, T1-slope was significantly correlated to T1-tilt ($r = 0.549$, $p = 0.002$), to the thoracic kyphosis ($r = 0.496$, $p = 0.005$) and to the lumbar lordosis ($r = 0.433$, $p = 0.017$). Furthermore, changes in cervical lordosis were significantly correlated to T1-slope changes ($r = 0.456$, $p = 0.013$) and thoracic kyphosis changes ($r = 0.354$, $p = 0.045$).
*Conclusions*: According to these results, T1 is of crucial importance in order to evaluate sagittal alignment changes after AIS surgery. T1-slope and T1-tilt are valuable parameters for evaluation of regional (cervical lordosis and thoracic kyphosis) and global sagittal parameters. Furthermore, as these parameters are angular values, they are independent from the size and calibration of X-rays. T1 parameters represent for us crucial measurements for sagittal evaluation of AIS patients. However further studies on larger cohorts and correlation with clinical scores will be needed in order to confirm these results.
*Significance*: Evaluation of sagittal parameters is of primary importance. Among these parameters T1-slope is highly correlated with changes in regional curves and global alignment.

**OP43**/09:10–09:20

## New emerging technologies for the treatment of early onset scoliosis: a preliminary study

**David Eduard Lebel**[1], Shlomo Wientroub[2], Dror Ovadia[1]

[1]Dana-Dwek Childrens Hospital, Tel Aviv, Israel; [2]Tel Aviv Medical Center, Tel Aviv, Israel

**LEVEL 4/Spine**
Keywords: Early onset scoliosis

*Purpose*: Early onset scoliosis imposes challenges for the patient and surgeon.

Recently, new technology has evolved and might partially reduce the need for repetitive surgeries. The Magnetic expandable rods hold the promise for physiological growth without the need for distraction surgeries.

The purpose of this study was to describe the short-term experience with this device.
*Methods*: A retrospective review of patient's charts operated using the MAGEC® (magnetic expansion control) devise was undertaken. The device was inserted through a posterior approach. A brace was used for the first 4 months after index surgery. Distractions were started 2 months after index surgery and then every 2 months.
*Results*: 15 patients were treated with the system. All were diagnosed with early onset scoliosis. Five were with idiopathic scoliosis, nine with neuromuscular or syndromic scoliosis and one with congenital scoliosis. The average age at index surgery was 7.95 years (4.8–9.8). The mean Cobb angle measured 71° (56–90) and the average Cobb angle on first postoperative X-rays was 28.27° (12–45) with an average correction of 60 %. We followed the patients 3–20 months with minimum of one distraction and maximum 8 of distractions.

Two patients were revised due to proximal screw dislodgment which was attributed to misplacement at the index surgery. No complications occurred due to failure of the device.
*Conclusions*: Magnetic growing rods for early onset scoliosis are safe and save the patients from repeated distractions under general anesthesia.
*Significance*: This new device was evaluated for safety.

**OP44**/09:20–09:30

### Radiological and functional outcomes of Lenke 5 adolescent idiopathic scoliosis treated by selective posterior fusion with a minimum 5-year follow-up

**Brice Ilharreborde**, Jihane Rouissi, Keyvan Mazda

Robert Debré Hospital, Paris, France

**LEVEL 4/Spine**
Keywords: Adolescent idiopathic scoliosis, Lenke 5

*Purpose*: Most of the studies of the outcomes of Adolescent Idiopathic Scoliosis (AIS) patients report only 2-year follow-up. Lumbar and thoracolumbar curves very often require a distal fusion ending on L3 or L4, with only 2 or 3 lumbar mobile segments remaining. They are, therefore, potentially candidates for early adjacent degenerative change. The aim of this study was to report the radiological and functional outcomes of Lenke 5 AIS treated by selective posterior fusion, with a minimum 5-year follow-up.
*Methods*: 40 patients (mean age 15.5 years) were included. Mean follow-up was 8 years (5–10.5 years). Frontal and sagittal spinal and pelvic parameters were measured using EOS low-dose stereoradiography. The surgical corrections were calculated postoperatively and at latest follow-up. The aims of surgery were to obtain a postoperative frontal imbalance <2 cm, a remaining ilio-lumbar angle <5° and a restored sagittal alignment. Adjacent complications, such as proximal junctional kyphosis, progression of the unfused thoracic curve and distal adding on were reported. In addition, lumbar disc heights were measured at each evaluation, and SRS-22 scores were obtained at latest follow-up.
*Results*: There was a statistically significant improvement of the mean coronal lumbar Cobb angles ($p < 0.001$), with no loss of correction at follow-up. Postoperative imbalance was significantly reduced after surgery, and progressive improvement was observed during follow-up. L1/S1 lumbar lordosis averaged 60° at follow-up, but only 50 % of the patients had a lordosis adapted to their pelvic incidence (i.e. LL-PI <9°), with a tendency towards overcorrection. The mean ilio-lumbar angle was corrected from 14° to 5° postoperatively, but 42.5 % of the patients still had a remaining angle >5° at follow-up, without significant influence on the SRS score. T4 frontal tilt remained stable over time. Average SRS-22 score at follow-up was 4.06, with no correlation to any radiological parameter. No progression of the unfused thoracic curves was observed, but 8 patients (20 %) developed radiological proximal junctional kyphosis (PJK). However, only 2 patients (5 %) required revision. L3/L4 and L4/L5 disc heights increased after surgery, and remained unchanged at follow-up. One patient developed symptomatic distal adding-on and required 2 revisions (2 years and 6 years postoperatively). The overall revision rate was 15 %, and 73.5 % of the patients reported no use of pain medication.
*Conclusions*: Radiological and functional outcomes of Lenke 5 AIS treated by posterior selective fusion are satisfactory at 8-year follow-up, even in fusions extending to L4. Only 7.5 % underwent revision for adjacent complications, and the L4/L5 disc height remained stable over time. However, the correlation between radiological parameters and SRS-22 scores needs to be further studied at a minimum 10-year follow-up.
*Significance*: This study is the first one to provide radiological and functional outcomes at 8-year follow-up of Lenke 5 AIS treated by selective segmental posterior fusion

**OP45**/09:30–09:40

### Sagittal spino-pelvic organization influences the biomechanical behavior of the intervertebral disc after idiopathic scoliosis surgery: a prospective study with minimum 2 years follow up

**Kariman Abelin-Genevois**[1], Pascal Swider[2], Erik Estivalezes[2], Jerome Sales De Gauzy[3]

[1]Hopital femme Mere enfant Université Claude Bernard Lyon I, Lyon, France; [2]Laboratoire de Biomécanique UMR CNRS 5502, Toulouse, France; [3]Children's Hospital, Toulouse, France

**LEVEL 2/Spine**
Keywords: Idiopathic scoliosis, Intervertebral disc, Spino pelvic congruity, Pelvic incidence

*Purpose*: To analyse the biomechanical properties of the intervertebral disc after scoliosis surgery according to the underlying spino-pelvic organization.
*Methods*: We conducted a prospective MRI study in 45 patients with AIS with a minimum 2 years follow-up. Mean age at surgery was 15.1 years. Mean Cobb correction was 63 %. Fusion extended to L1 or L2 in 27 patients and to L3 or L4 in 18 patients. Total disc and nucleus volumes were extrapolated from 3D reconstruction using a custom-made image processing software (Biomechlab®, Toulouse, France). Nucleus and external disc contours were semi-automatically detected on turbo spin echo T2 weighted sequences (3 mm sagittal cuts).

Disc hydration was defined as the nucleus-disc volume ratio. Sagittal parameters were measured pre- and post-operatively on full spine standing views (pelvic incidence (PI), sacral slope, L1/S1 lumbar lordosis). Lumbar-pelvic congruity was represented by LL/SS ratio as described by Stagnara.
*Results*: Mean PI of the cohort was 55 (34–80). In low PI group ($n = 24$), mean loss of lordosis was 3° (NS). Lumbo-pelvic congruity was reduced from 1.53 to 1.27 ($p = 0.01$). Disc volumes remained stable more than 2 years after the surgical correction. Significant increase of the nucleus volume and disc hydration level occured at the latest follow-up at each level. The highest changes concerned the intermediate levels (+25 %, $p = 0.01$). Longer follow-up (5 years, $n = 13$) confirmed the constant increase of disc hydration level, especially when selective fusions were performed (from +25 to 39 % at L5S1 level, $p < 0.001$).

In the high PI group ($n = 21$), disc volumes and nucleus size remained unchanged after the surgery. Lumbo-pelvic congruity was not modified after surgery (LL/SS = 1.46).
*Conclusions*: Pelvic orientation had a significant influence on the biomechanical behavior of the free motion lumbar spine after spinal fusion in AIS. Lumbo-pelvic parameters positively influenced the post operative hydration properties when PI was in the lower range. These results can be explained by reduced constraints in the flat sagittal profile. When PI is high, shear stresses maintain disc homeostasis disturbances under the fusion mass, whatever the coronal correction outcome. Pelvic morphology plays an important role in the biomechanical behavior of intervertebral discs after scoliosis surgery. Patients with high pelvic incidence may present a higher risk for accelerated disc degenerative changes after extended fusion.
*Significance*: This prospective MRI study in AIS patients showed a significant and sustainable improvement in disc hydration properties after surgery. These changes are clearly under the influence of the sagittal spino-pelvic organization.

**OP46**/09:40–09:50

## The development of the ischio-iliac lordosis during growth as an essential adaptation towards human bipedalism and a determinant of sagittal balance

**Tom P Schlosser**[1], Michiel M. Janssen[1], Tomaz Vrtovec[2], Franjo Pernus[2], F. C. Oner[1], Max A. Viergever[3], Koen Vincken[3], René M. Castelein[1]

[1]Department of Orthopaedic Surgery, University Medical Center Utrecht, Utrecht, The Netherlands; [2]Faculty of electrical engineering, University of Ljubljana, Ljubljana, Slovenia; [3]Image Sciences Institute, University Medical Center Utrecht, Utrecht, The Netherlands

**LEVEL 2/Spine**
Keywords: Ischio-iliac lordosis, Sagittal spino-pelvic alignment, Normal growth

*Purpose*: The development of a lordosis between the ischium and ilium distinguishes humans from all other mammalians, because it allows ambulation in a unique upright bipedal manner while forceful extension of the legs is preserved. The importance of sagittal pelvic morphology has been recognized in a number of spinal conditions, most notably idiopathic scoliosis and spondylolisthesis. Nevertheless, lordotic angulation between ischium and ilium has received little attention. This is the first study to define and quantify the lordotic angulation between the ischium and ilium in the normal growing and adult spine and to evaluate its correlation with the widely described pelvic inclination.
*Methods*: A consecutive series of three-dimensional computed tomography scans of the abdomen of asymptomatic children ($n = 199$) and adults ($n = 310$) were used. Scan indications were trauma screening or acute abdominal pathology. Using previously validated image processing techniques, the femoral heads, center of the sacral endplate and the axes of the ischial bones were semi-automatically identified. A true sagittal view of the pelvis was automatically reconstructed, on which ischio-iliac angulation and pelvic inclination were calculated. The ischio-iliac angle was defined as the mean angle between the axes of the ischia and the line from the midpoint of the sacral endplate to the center of the femoral heads.

*Results*: A wide natural variation of the ischio-iliac angle (3°–46°) and pelvic incidence (14°–77°) was observed. Pearson's analysis demonstrated a significant correlation between the ischio-iliac angle and pelvic incidence ($r = 0.558$, $P < 0.001$). Linear regression analysis revealed that ischio-iliac angle, as well as pelvic incidence, increases significantly during childhood (+7° and +10°, $P < 0.001$, respectively) and becomes constant after adolescence.
*Conclusions*: Increasing ischio-iliac angle and pelvic inclination during normal pediatric growth appear as essential adaptations for a well-balanced adult spine.
*Significance*: As the ischio-iliac angle and pelvic inclination reflect the development of the sagittal profile of the spine, their role in the development of certain spinal pathologies may be worthy of further study.

**OP47**/09:50–10:00

## Natural sagittal spino-pelvic alignment in boys and girls before, at and after the adolescent peak height velocity

**Tom P. Schlosser**[1], Suken A. Shah[2], Kenneth Rogers[2], Koen L. Vincken[3], René M. Castelein[1]

[1]Department of Orthopaedic Surgery, University Medical Center Utrecht, Utrecht, The Netherlands; [2]Department of Orthopaedic Surgery, Nemours/Alfred I. duPont Hospital for Children, Wilmington, USA; [3]Image Sciences Institute, University Medical Center Utrecht, Utrecht, The Netherlands

**LEVEL 2/Spine**

Keywords: Sagittal spino-pelvic alignment, Normal growth, Posteriorly directed shear forces

*Purpose*: One of the unexplained but well known characteristics of adolescent idiopathic scoliosis is that girls are far more often affected than boys and that the onset and progression of the deformity normally occurs around the adolescent growth spurt. It has previously been shown that rotational stiffness of the spinal segments is decreased by posteriorly directed shear loads. These shear loads uniquely act on the human spine and might play an important role in the onset of rotational spinal deformities, as in idiopathic scoliosis. It can be inferred that certain sagittal spinal profiles are more prone to develop a rotational deformity that may lead to idiopathic scoliosis. In this international multicenter study, sagittal spino-pelvic alignment and inclination of each individual vertebra in normal boys and girls will be quantified before, at and after the peak of the adolescent growth spurt.
*Methods*: Standardized lateral radiographs of the spine of children ($n = 148$) who underwent scoliosis screening and had a normal spine were included in this study. Children with spino-pelvic pathology, neuromuscular disease, syndromes associated with disorders of growth or incomplete visualization of the full spine and femoral heads were excluded. As suggested by Dimeglio, subjects were classified according to the timing of the peak of the growth spurt, based on skeletal characteristics, closure of the triradiate cartilage and Risser's stage, into three cohort: before ($n = 44$; mean age girls, $9.9 \pm 1.4$ years; boys, $10.7 \pm 1.8$), at ($n = 38$; girls, $12.3 \pm 1.1$; boys, $13.7 \pm 0.7$) and after ($n = 45$, girls, $14.0 \pm 1.4$; boys, $14.8 \pm 1.4$) the peak of the growth spurt. A systematic study using a semi-automatic measurement of nine sagittal spino-pelvic parameters, describes the backwardly inclined segment and inclination of each individual vertebra between C7 and L5. This was performed for each subject using an in-house developed computer software.
*Results*: Thoracic kyphosis, pelvic inclination and pelvic tilt were smaller and the backwardly inclined segment longer before and at the peak of the growth spurt compared to after the growth spurt ($P < 0.001$, $P = 0.023$, $P = 0.007$ and $P < 0.001$, respectively). In addition, relatively steeper backward inclination of T1–T8 was observed before and at the growth spurt, compared to after the growth spurt. No differences in global sagittal parameters were observed between boys and girls, however, the backwardly inclined segment was significantly longer ($P = 0.001$), and T3–T11 were steeper backwardly inclined in girls compared to boys ($P < 0.05$).
*Conclusions*: The result imply that before and at the peak height velocity, especially in girls, spinal segments are more backwardly inclined and affected by greater posteriorly directed shear loads.
*Significance*: This may explain why the onset and progression of adolescent idiopathic scoliosis frequently occurs in girls in the beginning of the adolescent growth spurt.

**April 4th**
Spine Part II
10:30–11:20
CONCERT HALL
**OP48**/10:30–10:40

### Sublaminar band: are they safe?

**Eva Polirsztok**, Martine Gavaret, Thibault Gsell, Marie-Christine Maximin, Gerard Bollini, Jean-Luc Jouve

La Timone Enfants, Marseille, France

**LEVEL 4/Spine**
Keywords: Sublaminar band, Neurophysiologic monitoring, Anesthesiologic monitoring

*Purpose*: Sublaminar bands (SB) are now frequently used implants for spine deviation correction. Our purpose is to demonstrate their safety with a large series of patients.
*Methods*: This is a retrospective study. We treated in our department 378 spine deviations in children and adolescents with a hybrid posterior technique (lumbar screws, hooks and thoracic SB). Each surgery was managed with an anesthesiological and neurophysiological monitoring (SSEP and NMEP). An alert was described as an amplitude decrease of 50 % or a latency increase of 10 %. Data were analyzed using Student test or Wilcoxon test.
*Results*: We used 2,223 SB in 378 operative procedures. We described 10 neurophysiologic alerts, due to the passage of the band beneath the lamina. There is no significant difference regarding age and severity of the deformation between these two groups ($p > 0.05$). Neurophysiologic alert was always associated with a dysautonomic problem (hypertension and bradycardia). The lesional level was determined with a spinal electrode. In 6 cases, the responsible SB was removed. Three patients had post-operative neurologic deficiency (0.8 %) one without complete recovery (localized incomplete sensory deficiency). In the same series of 378 patients, 21 alerts were reported due to a screw or a hook or during the correction maneuver, without dysautonomic trouble.
*Conclusions*: The SB neurologic complications rate is as high as other implants. Simultaneous hemodynamic and neurophysiological change is an argument for vegetative response due to SB passage. Their optimal use requires strict learning process for their insertion beneath the lamina in order to be as least traumatic as possible.
*Significance*: The SB is as safe as any other spine implants.

**OP49**/10:40–10:50

### Role of thoracoscopic anterior release for sagittal correction in hypokyphotic AIS

**Emmanuelle Ferrero**[1], Brice Ilharreborde[1], Benjamin Blondel[2], Jean-Luc Jouve[2], Keyvan Mazda[1]

[1]Robert-Debre Hospital, Paris, France; [2]CH la timone, Marseille, France

**LEVEL 4/Spine**
Keywords: Adolescent idiopathic scoliosis, Hypokyphosis, Thoracoscopy

*Purpose*: Thoracic Adolescent idiopathic scoliosis (AIS) curves (Lenke 1–4) are often characterized by hypo-kyphosis between T4 and T12, which has been associated with impairment of pulmonary function. Sagittal alignment remains challenging to correct, even with recent posterior segmental instrumentation. Some authors suggested anterior endoscopic release (AER) to reduce the anterior column height, and, therefore, ease the thoracic kyphosis correction. The aim of this study was to assess the influence of AER on sagittal correction in hypokyphotic AIS.
*Methods*: 56 hypokyphotic (T4/T12 <20°) AIS patients were included. In Group 1 (28 patients), patients first underwent AER, followed by posterior segmental instrumentation and correction 5–7 days later. In Group 2 (28 patients), patients were treated by a single posterior procedure. Posterior correction was performed in all cases using postero-medial translation, using hybrid constructs, combining lumbar pedicle screws and thoracic sublaminar bands. A minimum 2-year follow-up was required. Radiological measurements were performed using low dose stereoradiography (EOS system), and the thoracic kyphosis correction was compared between the 2 groups.
*Results*: Both groups were comparable regarding demographic data and preoperative thoracic kyphosis (group 1: $11.7° \pm 6.9$ vs group 2: $12.1° \pm 6.3$, $p = 0.89$). Postoperative thoracic kyphosis increase averaged $18.3° \pm 13.6$ in Group 1 and $15.2° \pm 9.0$ in Group 2. At latest follow-up, 75 % of the patients where normokyphotic in Group 1 and 85 % in Group 2. The benefit of the anterior release was not statistically significant ($p = 0.35$). The mean length of hospitalization stay was 5 days longer in Group 1 due to the treatment protocol, but no complication related to the anterior release was reported.
*Conclusions*: Thoracoscopy anterior release remains a safe and reliable technique that can be considered in stiff scoliotic curves to emphasize the frontal correction. However, results of the current series show that no additional significant benefit can be expected in the sagittal plane when a posteromedial translation technique with sublaminar bands is used for correction, even in challenging hypokyphotic patients.

*Significance*: Thoracoscopic anterior release is not necessary to improve sagittal correction in thoracic AIS patients treated by posteromedial translation.

**OP50**/10:50–11:00

## Mortality and radiographic outcomes in severe scoliosis of 90° or more. A comparison of hybrid with total pedicle screw instrumentation

**Ilkka Helenius**[1], Tuomas Jalanko[2], Mikko Mattila[2]

[1]Department of Pediatric Orthopaedic Surgery, Turku, Finland; [2]Hospital for Children and Adolescents, Helsinki, Finland

**LEVEL 2/Spine**

Keywords: Severe scoliosis, Hybrid instrumentation, Pedicle screw instrumentation, Mortality

*Purpose*: Untreated severe scoliosis is associated with increased mortality and remains a significant surgical challenge. Few studies have reported mortality after surgical treatment of severe scoliosis beyond a 2 year follow-up and/or health related quality of life.

*Methods*: We evaluated 32 consecutive patients [11 males, mean age at surgery 15.3 (range 10.7–20.7) years] operated for a scoliosis of 90° or more using either Hybrid (H, n = 15) or Pedicle Screw (PS, n = 17) instrumentation. Follow-up time averaged 2.9 (2.0–6.6) years for radiographic and quality of life measurements and 5.5 years (2.0–9.0) years for mortality data. Of them, one had adolescent idiopathic scoliosis, three secondary scoliosis, and 28 neuromuscular scoliosis. Twelve patients in the H and two patients in the PS group underwent antero-posterior surgery ($p < 0.001$) and three patients in both groups had an apical vertebral column resection.

*Results*: One (3.1 %) patient died during follow-up from severe pneumonia. Preoperatively, the mean magnitude of the major curve was 109° (90°–127°) in the H and 100° (90°–116°) in the PS group ($p = 0.015$). This was corrected to 45° (19°–69°) in the H and 27° (18°–40°) in the PS group at 2-year follow-up ($p < 0.001$) with a mean correction of the major curve of 59 % (37–81 %) in the H vs. 73 % (60–81 %) in the PS group, respectively ($p = 0.0023$). There were six postoperative complications including one transient spinal cord deficit necessitating reoperation in the H group as compared with five complications in the PS group ($p = 0.53$). The SRS-24 total score averaged 97 (64–108) points in both groups at 2-year follow-up with no significant difference between the study groups.

*Conclusions*: Mortality rate after surgical treatment of severe scoliosis seems acceptable. Severe scoliosis can be treated safely with significantly better correction of the spinal deformity using pedicle screws than hybrid instrumentation.

*Significance*: Mortality rate was low up to a mean of 5 years follow-up. Total pedicle screw instrumentation provided shorter operative time, somewhat less blood loss, better major curve correction with less need for antero-posterior surgery as compared with hybrid constructs in patients with severe scoliosis.

**OP51**/11:00–11:10

## Low infection rates using fresh-frozen allograft in spine surgery. An analysis of 206 consecutive deformity cases

**Dietrich Schlenzka**, Timo Yrjonen, Timo Laine, Teija Lund, Heikki Osterman, Mikko Poussa

ORTON Orthopaedic Hospital, Helsinki, Finland

**LEVEL 4/Spine**

Keywords: Spinal deformity, Surgery, Allogeneic bone, Infection

*Purpose*: The purpose of the study was to evaluate the safety of fresh-frozen allograft in spine surgery with special attention to postoperative infection.

*Methods*: The study is based on the prospective bone bank registry and the infection registry of the institution.

*Results*: From 1985 to 2009, allogeneic bank bone chips from fresh frozen femoral heads were used in 309 patients. Of these, 206 had primary posterior deformity surgery with instrumentation. Clinical records were available from all 206 patients. There were 121 females and 86 males, mean age 16.6 years (8–51). The indications for surgery were: CP 48, Idiopathic scoliosis 37, Psychomotoric retardation 31, Duchenne's 19, Scheuermann's 10, MMC 10, Spinal muscular atrophy 6, syndromes and rare diagnoses 45.

The following instrumentation systems were used: Harrington-Luque 87, Double-rod hybrid 81, Double rod hybrid with sublaminar wires 27, others 11. The number of vertebrae included was 12.9 (5–21). Bone chips of 5–10 mm diameter were applied to the fusion bed. The bone was not washed after unfreezing; no local antibiotics were administered. The mean operation time was 241 min (120–515), the mean intraoperative blood loss 1,644 ml (200–8,000). One-hundred-and-ninety-four patients

(94.5 %) received prophylactic i.v. antibiotics during induction.

One patient died at the third postoperative day from respiratory complications. Thus, 205 remaining patients could be followed for mean 47.7 months (6–252). Two patients were lost resulting in a follow-up rate of 99.0 %.

The mean stay in hospital was 12 days (6–95). Postoperative body temperature >37.5 °C was measured for mean 3 days (1–14) in 170 (82.9 %) patients. Additional antibiotic treatment was given in 82 (40 %) patients. The main indications were respiratory infection 25, UTI 28, and wound problems 12. According to the clinical records and the infection registry of the hospital, there were three early deep wound infections (1.5 %) and 11 (5.3 %) superficial infections. All three deep infections and two superficial infections were seen in MMC patients who developed skin necrosis in the lumbosacral area. All five healed after plastic surgery. The implants could be saved. The remaining 9 superficial infections healed after short courses of antibiotic treatment (7 patients) or local treatment (2 patients) without antibiotics. No late infections were found.

There were 20 other reoperations: implant prominence and pain 7, balance problems 4, pseudarthrosis 3, hook dislodgement 3, rod breakage 2, neurology 1.

*Conclusions*: Fresh frozen allograft used in the way reported by the authors seems to be a safe option for posterior spinal fusion. The infection rate was unexpectedly low taking into account that the great majority (82 %) of the patients had neuromuscular or syndromic disorders.

*Significance*: The additional trauma, operation time, and blood loss due to harvesting of patients' own bone could be avoided.

**OP52**/11:10–11:20

### A computed tomography-based study on asymmetry and intrinsic torsion of the intervertebral discs versus vertebral bodies in adolescent idiopathic scoliosis

**Tom P Schlosser**[1], Rob C. Brink[1], Marijn Van Stralen[2], Winnie C. Chu[3], Tsz P. Lam[4], Koen L. Vincken[2], René M. Castelein[1], Jack C. Cheng[4]

[1]Department of Orthopaedic Surgery, University Medical Center Utrecht, Utrecht, The Netherlands; [2]Image Sciences Institute, University Medical Center Utrecht, Utrecht, The Netherlands; [3]Department of Diagnostic Radiology and Organ Imaging, Prince of Wales Hospital, Chinese University Hong Kong, Shatin, Hong Kong SAR; [4]Department of Orthopaedic Surgery and Traumatology, Prince of Wales Hospital, Chinese University Hong Kong, Shatin, Hong Kong SAR

**LEVEL 2/Spine**

Keywords: Adolescent idiopathic scoliosis, Intervertebral disc, Asymmetry, Intrinsic torsion

*Purpose*: On conventional two-dimensional radiographs of adolescent idiopathic scoliosis (AIS) patients the intervertebral discs (IVDs) are more wedge shaped than the osseous vertebrae. In addition it has been demonstrated that the content of the IVDs in AIS patients is abnormal. After more than a century of dedicated research, the aetiology and pathogenesis of AIS is still not completely understood. According to the 18th and 19th century anatomists, however, it was already demonstrated that AIS is a three-dimensional deformity of the spine, characterized by lateral deviation, axial rotation and lordosis. The two-dimensional results suggests that the morphological changes of the spine in AIS may primarily be caused by morphological changes of the IVDs. This is the first study evaluating the coronal and sagittal asymmetry as well as the intrinsic torsion of the vertebrae versus the IVDs in AIS.

*Methods*: A unique series of high-resolution CT scans of 88 AIS patients (mean main thoracic Cobb, 59°; thoracolumbar/lumbar, 37°) that underwent CT guided pedicle screw placement was used for this study. Ten atypical AIS curves were excluded because patients had neurological symptoms or left convex curves. Based on 3-D segmentation of each individual endplate between T1 and L5, semi-automatically, the longitudinal axis and true sagittal section were reconstructed for all IVDs and vertebrae and the morphological parameters calculated. Relative anterior-posterior and left–right asymmetry (% length asymmetry) and intrinsic torsion (degrees axial torsion/cm height) were compared between the IVDs and vertebrae in the apical and non-apical regions of the main thoracic and thoracolumbar/lumbar curves.

*Results*: There was on average at least three times more left–right and anterior-posterior asymmetry in the IVDs than in the osseous vertebrae, in the main thoracic as well as the thoracolumbar/lumbar curves ($P < 0.001$). Moreover, while the mean torsion of the thoracic vertebra was 2°/cm height, the torsion of the IVDs was 6°/cm ($P < 0.001$). While anterior-posterior and right-left asymmetry of the IVDs and vertebrae was greater in the apical segments compared to the non-apical segments, torsion was most pronounced in the non-apical levels ($P < 0.001$).

*Conclusions*: This study confirms that the deformity in AIS is mainly a deformity of the IVDs.

*Significance*: Because of the cross-sectional design of the study, it cannot be derived whether the deformity in the IVD is the primary cause of AIS, or it is secondary to abnormal mechanical forces applied to the IVD.

**April 4th**
Limb Reconstructions
11:20–12:30
CONCERT HALL
**OP53**/11:20–11:30

### Limb lengthening in patients with rhizomelic dwarfism: a comparative study of femoral and tibial lengthening

**Kwang-Won Park**, Hae-Ryong Song

Korea University Rare Diseases Institute, Seoul, Korea

**LEVEL 4/Limb reconstruction**
Keywords: Limb lengthening, Rhizomelic dwarfism, Achondroplasia

*Purpose*: Achondrodysplasia is the most common form of rhizomelic dwarfism. Bilateral lower limb lengthening has been performed on these patients. However, the tibia and femur were not compared with each other as far as the rate of callus formation, consolidation and complications were concerned. Our study aims to answer whether there really is rhizomelia in achondroplasia and to compare femoral and tibial lengthening in the same set of patients by comparing their respective callus features, other certain radiographic parameters, clinical results and complications.
*Methods*: A total of 32 femoral and 32 tibial lengthenings performed on 16 patients with achondroplasia in our institution from 2004–2012 were included in a retrospective review. We measured the pixel value ratio (PVR) of the lengthened area on radiographs and each radiograph was analysed for the shape, type and density of the callus. The external fixation (EFI) and healing indices (HI) were also computed to compare both tibial and femoral lengthening.
*Results*: All procedures achieved the planned lengthening. In the tibia, the PVR, EFI and HI were all significantly better than that of the femur. Femoral lengthening was associated with an increased incidence of concave, lateral and central callus shapes. Tibial lengthening had a lower complication rate than femoral lengthening. Callus formation in the tibia during the distraction period proceeded at a significantly higher rate than that in the femur.
*Conclusions*: These findings suggest that bilateral limb lengthening, tibia first then the femur, is still a reasonable option for patients with achondroplasia, provided the patients are carefully monitored for complications and they are promptly addressed whenever they occur.
*Significance*: In bilateral limb lengthening for achondroplasia patients, we should lengthen the tibia first because of lower complication rate and faster callus formation than femur.

**OP54**/11:30–11:40

### Is there a correlation between the interscrew angle and the joint orientation angles after hemiepiphysiodesis with the Eight plate?

**Salih Marangoz**[1], Kadir Buyukdogan[1], Sevilay Karahan[2]

[1]Hacettepe University, Faculty of Medicine, Department of Orthopaedic Surgery, Ankara, Turkey; [2]Hacettepe University, Faculty of Medicine, Department of Biostatistics, Ankara, Turkey

**LEVEL 4/Limb reconstruction**
Keywords: Hemiepiphysiodesis, Joint orientation angles, Eight plate, Interscrew angle, Mechanical lateral distal femoral angle, Medial Proximal tibial angle

*Purpose*: The screws of the Eight plates are inserted at a certain angle which never stays the same throughout the correction. We wanted to investigate if there was any correlation between the so-called interscrew angle (ISA), and the joint orientation angles (JOA), namely, mechanical lateral distal femoral angle (mLDFA) and medial proximal tibial angle (MPTA).
*Methods*: A retrospective medical chart review was conducted. Patients who were treated consecutively for genu valgum or genu varum by means of hemi-epiphysiodesis with an Eight plate were included in the study. Applications of double plate hemi-epiphysiodesis of the same hemiphysis or simultaneous applications of the knee and ankle were excluded. All diagnoses and ages were included in the study, because the hypothesis was that there would be a correlation at all times. Pre-operative long leg X-rays, immediate post-operative AP X-rays of the tibia or femur, and follow-up long leg X-rays were used for measurements. The ISA was defined as the angle between the long axis of the screws on each side of the physis of the eight plate construct. Measurements were made by two investigators. Immediate post-operative and final follow-up ISAs were measured and the difference was analyzed in comparison to the pre-operative and final follow-up JOAs in order to find a correlation using Pearson correlation coefficient. Regression analyses were performed while investigating the effect of ISA on the JOA. A power analysis was then initiated with the resultant data.
*Results*: The chart review revealed 53 segments of 30 consecutive patients to be included in the study. There were 29 femoral and 24 tibial applications in 18 male and 12 female patients. The mean age of the patients was 9 (range 4–16). The diagnoses included, but are not limited to, fibular hemimelia, metabolic bone diseases, Blount's disease, Cozen phenomenon, multiple hereditary exostosis, and idiopathic. Preoperative mean mLDFA corrected from 79° to 91.9° at the final follow-up and preoperative mean MPTA corrected from 100.5° to 92.1° at the final follow-up. Mean ISA in the femur changed from 12° to 32.3°, whereas in the tibia it changed from 8.3° to 25.4°. A high level of correlation was found. Regression analyses resulted with formulas that might predict the angular change. Power analysis yielded 96.3 %.
*Conclusions*: There is a high correlation between the ISA and the JOA. This correlation is not dependant on the age or the diagnosis of the patient. The ISA follows the JOA at a predictable amount through formulas which regression analysis suggest.
*Significance*: This study confirms the clinical observation of the diverging angle between the screws is correlated with the correction of the JOA. It also gives formulas to be able to predict the amount of change necessary to achieve the desired correction in the JOA.

**OP55**/11:40–11:50

## Femoral lengthening with a motorized intramedullary nail—a matched pair comparison with Ilizarov external lengthening

**Joachim Horn**, Harald Steen

Oslo University Hospital, Rikshospitalet, Oslo, Norway

**LEVEL 3/Limb reconstruction**
Keywords: Limb lengthening, Ilizarov, Motorized nail

*Purpose*: Lengthening with a fully implantable motorized and remote controlled intramedullary nail is a relatively new method. The aim of our study was to see if an intramedullary lengthening device might avoid problems which are normally associated with the Ilizarov technique, and to see if it is a reliable device for limb lengthening and deformity correction.
*Methods*: 26 femoral lengthenings were included in this study. We performed a retrospective matched-pair comparison of 13 femoral lengthenings (13 patients) with a motorized intramedullary nail (Fitbone) versus 13 femoral lengthenings (13 patients) with an external ring fixator (Taylor's Spatial Frame). The patients were matched based on age, gender, amount of lengthening, and the aetiology of leg length discrepancy. Outcome measures were consolidation index, the success of the procedure in terms of achieved lengthening and alignment, joint range of motion (ROM) and complications. Mean leg length discrepancy for all patients was 37 (15–75) mm.
*Results*: The full amount of lengthening was achieved in all but 1 patient in each group. 5 patients in the Ilizarov group and 1 patient in the Fitbone group had a small residual deformity at the end of treatment. The consolidation index for the patients lengthened with a Fitbone nail was 1.4 (0.9–2.5) months/cm and for the Ilizarov group 2.5 (0.9–2.75) months/cm. Based on the Paired samples *t* test, this difference was statistically significant ($p = 0.012$). In the Fitbone group almost all patients had full extension and at least 90 degrees of flexion in the knee during the lengthening period. 6 weeks after completed lengthening almost all patients with nail lengthening had regained full ROM. In the Ilizarov group ROM in the knee during lengthening was significantly reduced with a mean flexion ability of 25 (5–60)°. 6 months after frame removal 5 patients had still less than 90 degrees of flexion, 3 patients required open (Judet procedure) and 2 patients closed mobilization of the knee. There were 3 minor complications in the Fitbone group and 2 major complications in the Ilizarov group.
*Conclusions*: Both techniques allow controlled lengthening and deformity correction. Consolidation of the regenerate was somewhat faster in the Fitbone group. However, the most important difference between these two lengthening techniques is the rehabilitation of the patients with maintenance of knee movement.
*Significance*: The results of this investigation suggest that difficulties usually associated with the use of external fixation, can be avoided when using a motorized nail. Sufficient lengthening and axis correction can be achieved with both techniques.

**OP56**/11:50–12:00

## Congenital absent tibia

**Gamal Ahmmed Hosny**

Benha Faculty of medicine, Cairo, Egypt

**LEVEL 4/Limb reconstruction**
Keywords: Congenital absent tibia, No amputation, Treatment

*Purpose*: Congenital absent tibia is a rare problem. The treatment is basically early amputation. However, due to the social pressure we prefer a conservative approach.
*Methods*: 69 cases with Congenital Tibial hypoplasia [2],Type Ia [25], Type Ib [6], Type II [31] and Type IV [5] cases using Jones classification were treated in our center. 6 cases were operated on both sides [75 limbs].There were 38 females. The age of patients at the first operation ranged from 1 to 20 years. In Type I there were 3 operative steps: (1) Gradual centralization using the fixator. (2) Centralization and Casting. (3) Correction of residual knee and ankle deformities using the fixator. Steps 1 and 2 were done concomitantly in 7 cases. In Type II and IV differential lengthening was performed with correction of the foot deformity. The femur was over-lengthened in 9 cases to compensate for the length inequality. The patients were evaluated clinically and radiographically regarding their functional knee activity, stability, pain, limb length inequality and satisfaction of the patient.

*Results*: There was functional improvement in all patients. There was residual knee instability in Type 1 cases. Follow up ranged from 2 to 14 years. The magnitude of lengthening ranged from 20 % till 100 % of the original length. Complications included: Pin track infection, knee flexion deformity in 13 cases, fractures of the fibula or regenerate or the femur in 8 cases.

*Conclusions*: Management of congenital absent tibia can be treated without amputation as functional improvement is expected despite the lengthy treatment and high rate of complications.

*Significance*: Treatment of congenital absent tibia without amputation is a valid option.

**OP57**/12:00–12:10

## How precise is the PRECISE®—a comparison between a new magnetic external motorized controlled lengthening device, and the not-available-anymore mechanical ISKD®—some implant associated problems are resolved, other problems are new

**Frank M. Schiedel**

Muenster University Hospital, Muenster, Germany

**LEVEL 2/Limb reconstruction**

Keywords: Intramedullary limb lengthening, External motorized magnetic lengthening device, PRECICE(R) nail, Comparison, Complications, Lower limb deformity correction

*Purpose*: Various devices are used for limb lengthening in patients with leg lengths discrepancies. The PRECICE® intramedullary limb lengthening system uses a magnetic rod for the extension of two nail parts. This needs a motorized external remote controller (ERC) with a rotational magnetic field for stimulation. The aim of this study is to evaluate the reliability and safety of the PRECICE system and to compare the preliminary results with the known difficulties in a case series with usage of a mechanical device (intramedullary skeletal kinetic distractor).

*Methods*: In a prognostic single-center short-term follow-up study PRECICE lengthening devices were implanted for leg lengthening. Follow-up was 6 months after consolidation or full weight-bearing. The modified Paley classification for evaluating problems, obstacles, and complications in bone lengthening was used to describe implant specific and ERC associated difficulties and to separate them from non-implant associated difficulties. These preliminary results were chosen to compare with the results of an already published cohort of 69 patients with ISKD®.

*Results*: Starting in June 2012 26 PRECICE nails were implanted in 24 patients. Two nails failed to lengthen. 24/26 nails lengthened over the desired distance, which represents a reliability of 92 %. Two nail breakages, one in the welding seam, were observed during consolidation. One other breakage was accidental. ERC usage was problematic mostly in the patients with femoral lengthening. In 10/24 successful patients further adjustment of the ERC was necessary. Problems were seen 15/24 cases, obstacles and complications each in 4/24 cases. The average desired lengthening was 38 mm, the average reached lengthening was 36.8 mm. This means an accuracy of 97 %.

*Conclusions*: Leg lengthening using the PRECICE shows a high rate of accuracy, particularly in the femur, with good reliability of the implant. In addition to the ISKD the thin magnetic extension rod, which can be damaged in different ways, is a weak point. The other difficulty seems to be the proper usage of the ERC in connection with the low magnetic field. With precise knowledge of these difficulties and well-designed patient information the usage of PRECICE is safe. Using the modified Paley classification, the results can be presented in a way that is comparable with external fixation procedures and other intramedullary procedures like the ISKD®.

*Significance*: The PRECICE(R) lengthening system is a new intramedullary external motorized magnetic limb lengthening device. The usage is safe and reliable. In comparison with known systems like the no longer available ISKD(R) there is no remarkable difference in the difficulties that one has to expect. Knowledge of some new aspects has highest significance for new users.

**OP58**/12:10–12:20

## Treatment of adolescent Blount's disease using a Taylor spatial frame with and without fibular osteotomy: is there any difference?

**Ofer Zaks**, Alexander Katzman, Mark Eidelman

Rambam Hospital, Haifa, Israel

**LEVEL 4/Limb reconstruction**

Keywords: Tibia vara, Blount, Fibular osteotomy

*Purpose*: To compare outcome of patients with adolescent Blount's disease treated with a Taylor spatial frame with and without fibular osteotomy.
*Methods*: 25 patients (27 tibias), 23 males and 2 females, mean age of 14.7 years (range 13–21 years) were included in our study. All patients underwent correction with Taylor spatial frame. Group A with fibular osteotomy, including 11 tibias and Group B with no fibular osteotomy including 14 tibias. Group A underwent correction by proximal tibial and fibular osteotomies (the fibula was fixed distally by two ilizarov wires to the distal ring). Group B was treated by proximal tibial osteotomy only, the fibula was not divided or stabilised).
*Results*: The goal of correction was achieved in 9 cases in group A and 12 in group B. Mean time in frame was 15.9 weeks in group A and 14.14 in group B. Mean lengthening was 16.5 mm in group A and 12.85 mm in group B. Mean proximal tibia-fibula distance was 20.45 mm (group A) and 14.9 mm (group B). Mean distal tibia-fibula distance was 9.81 mm (group A) and 9.64 mm (group B). There was no ankle malalignment in either Group. Complications included pin tract infection in 11 patients and delayed union in 2 patients (one in each Group).
*Conclusions*: We believe that correction of adolescent tibia vara may be performed safely without osteotomy of the fibula.
*Significance*: Based on our experience we did not find any significant difference between patients treated with and without fibular osteotomy in patients with adolescent Blount's disease.

**OP59**/12:20–12:30

## Tibial hemimelia rarely needs amputation

Mahzad Javid, **Gholam Hossain Shahcheraghi**

Namazee hospital, Shiraz, Iran

**LEVEL 4/Limb reconstruction**
Keywords: Lower extremity deformities, Congenital, Hemimelia, Tibia, Bone lengthening, Amputation

*Purpose*: The congenital absence of tibia, with occurrence of 1 per million live births, is a rare disease. One person alone does not often build up a large experience with its management. We would like to report our personal experience with 30 cases. We have undertaken limb salvage procedures in the majority of our cases, since amputation is strongly rejected by the families in our country.
*Methods*: 36 patients with 48 tibial hemimelia, who, had been under the direct care of the authors from infancy, were invited to come for clinical and radiologic evaluation. The patients or parents filled out a detailed questionnaire, which included, PedsQL (Pediatric quality of life), and parents' satisfaction. The tibial absence was classified according to both Weber and Jones classifications.

The full physical examination included: The foot, knee, and hip motion, leg alignment and length discrepancy; the speed, and pattern of gait, the type of shoe, brace or any walking aid used by the patients, the number of surgical interventions performed, with the complications and their effects on school attendance and absentees were also documented.
*Results*: From the total of 36 patients—17 girls and 19 boys, who had been under the care of the authors, 48 tibial-deficient limbs were evaluated. The mean age at final evaluation was 11 years and 6 months (2–23 years) and the mean follow up was 8 years (2–23 years). The 36 patients included 19 right, 5 left and 12 bilateral tibial hemimelias. The distribution of patients were as follows: 14 type I, 13 type II, 14 type IV, 7 unclassified (using the Jones classification); and 6 type I, 11 type II, 14 type III, 1 type IV, 2 type V, 14 type VII (Using the Weber classification).

Primary amputation was performed in 8 patients (9 limbs), and limb preservation surgeries were performed on 38 legs (26 patients). The most common ones included: Ilizarov lengthening (23 cases), centralization of ankle underneath the tibia (23 cases),and tibio-fibular synostosis (20 cases). The main complication was nonunion of the attempted synostosis between fibula and the remaining tibia which was seen in 3 patients (type II and IV hemimelia), and knee stiffness in 6 cases.

From the re-constructed limbs 18 are in regular shoes and 20 in short orthoses. Comparing the PedsQL in the group who had limb re-construction with the 8 cases who had amputation, there are a few points worth mentioning: walking scores, social and physical activities, and sporting were much better in the reconstructed group. The psychological scores and pain scores however were better in the amputated group. Patients and parents satisfaction scores were, similar in both groups.
*Conclusions*: Tibial hemimelia can be successfully reconstructed with a good functional outcome, with the use of Ilizarov lengthening in the majority of cases.
*Significance*: This is the largest series of tibial hemimelia, showing the high success rate in limb reconstruction and preservation.

**April 4th**
Cerebral Palsy/Foot Reconstruction
16:00–17:30
CONCERT HALL
**OP60**/16:00–16:10

### Longitudinal changes in foot posture in children with cerebral palsy

**Julieanne P. Sees**, Chris Church, Nancy Lennon, Joshua Schwartz, Tim Niiler, Daveda Taylor, John Henley, Freeman Miller duPont Hospital for Children, Wilmington, USA

**LEVEL 2/Cerebral palsy**
Keywords: Cerebral palsy, Foot

*Purpose*: Foot deformities are common in children with Cerebral Palsy (CP), yet the natural history has not been documented. The purpose of this study is to analyze longitudinal foot development and deformity in children with CP using a pedobarograph.
*Methods*: Ninety-six children with spastic CP were recruited in this Level II IRB approved prospective longitudinal study. Fifty-one children (16 unilateral, 35 bilateral involvement; 37 GMFCS I/II, 14 III/IV) who had a minimum of 5 visits were included in this analysis. The first visit was at age 33 ± 7 months and children were then evaluated every 6 months until age 5 years, and once a year there after until age 12 years (484 visits). We excluded data collected after boney foot surgeries, but not after soft tissue surgery. We compared this data to age-matched data from 366 feet of typically developing (TD) children. For each visit, the average of 3 footprints was analyzed. Outcome measures included the coronal plane pressure index (CPPI) and heel impulse. ANOVA with Tukey post hoc tests were used to compare the groups. Data were grouped and analyzed graphically using a thin plate spline analysis package for R statistical software.
*Results*: Variability in foot pressure data was higher in the children with CP vs. the TD group. From 3 to 10 years CPPI was higher (valgus) in GMFCS III/IV children compared to TD ($p < 0.05$).
*Conclusions*: The development of dynamic foot posture in walking is highly variable in children with CP especially in the early ages. Young children with CP tend to have a valgus foot distribution relative to TD. Valgus tends to persist in children with GMFCS levels III and IV and to normalize in children with GMFCS levels I and II.
*Significance*: Due to variability in the natural history of foot posture in children with CP, conservative management of coronal plane foot deformity is suggested, especially in young children and those ambulating without an assistive device.

**OP61**/16:10–16:20

### Should proximal femoral implants be removed routinely in children with cerebral palsy?

**Unni G. Narayanan**[1], Walter Truong[2], Tom Novacheck[2], Andrew Howard[1], Michelle Shin[1], Jasjit Lochab[3]

[1]The Hospital for Sick Children, Toronto, Canada; [2]Gillette Childrens Specialty Healthcare, St. Paul, Minnesota, USA; [3]University of Toronto, Toronto, Canada

**LEVEL 3/Cerebral palsy**
Keywords: Implant removal, Cerebral palsy

*Purpose*: The reasons reported for routine hardware removal in children include concerns for late infections, difficulty with later reconstructive surgeries, and fractures. However, there are concerns about costs and complications related to routine hardware removal. Non-ambulatory children with neuromuscular disorders are at increased risk. The aim of this study is to determine the complication rates and costs associated with the approach of a routine early removal of plates from the proximal femur in children with cerebral palsy (CP) with an approach of retention of implants and selective (reactive) removal. We hypothesize that the rate of complications related to implant retention is relatively low and would not justify routine removal of proximal femoral implants in children with cerebral palsy, given the risks involved with such a procedure.
*Methods*: Children ages 16 years or less with CP and proximal femoral hardware placement from 1991 to 2010 were categorized into two groups: (1) A routine early removal Group (within 2 years), and (2) a late selective removal/retained group. Patients were excluded if they had <2 years follow-up. Possible major complications were defined as fractures, deep infections, complications related to anaesthesia, and inability to completely remove implant. Patient risk factors for complications were defined and assessed in each group.
*Results*: 683 patients with CP and proximal femoral osteotomies were reviewed. 586 patients and 1,005 hips met criteria for this study. Average follow-up was 74 months (24–208). Group 1 included 291 patients and 517 hips while Group 2 included 295 patients and 488 hips. Both groups were similar in age, presence of seizure disorder, index procedure complications and length of follow-up. Group 2 was slightly older (8.5 vs 7.7) and slightly higher GMFCS level (4.0 vs 3.3). Major complication rates per patient were similar with Group 1 at 3.1 % (n = 9) and Group 2 at 4.7 % (n = 14) (P = 0.307). Early group complications included infection (5), fracture (3), aspiration pneumonia, C. Difficile infection (1). Late/retained group complications included infection (2), fracture (10), aspiration pneumonia (1), incomplete removal of implant (1). The average facility cost of proximal femoral hardware removal alone in children with CP was $25,525 in 2011. Total estimated Group 1 cost is $7,427,940 for routine removal alone in the 291 patients ($25,525/patient). 57 removals were eventually done in group 2. Including the estimated cost of treating the complications encountered in those 295 children, the estimated total cost is $1,695,754 ($5,748/patient).
*Conclusions*: In children with CP and proximal femoral hardware, there are similar complication rates when comparing routine early hardware removal at less than 2 years. There is an estimated savings of $20,000/patient, when plates are retained >2 years, rather than routinely removed.
*Significance*: This is the first comparative study to evaluate the practice of routine hardware removal with that of a strategy of implant retention and selective removal in response to symptoms or parental preferences.

**OP62**/16:20–16:30

### Head-shaft angle as predictor for hip displacement in children with cerebral palsy

**Gunnar Hagglund**[1], Maria Hermanson[1], Jacques Riad[2], Philippe Wagner[3]

[1]Department of orthopaedics, Lund, Sweden; [2]Department of orthopaedics, Skovde, Sweden; [3]National center for quality registers, Lund, Sweden

**LEVEL 2/Cerebral palsy**
Keywords: Cerebral palsy, Hip, Displacement, Dislocation, Head shaft angle

*Purpose*: To assess if the head-shaft angle (HSA) can predict hip displacement in children with cerebral palsy (CP).
*Methods*: CPUP (the Swedish follow-up program for CP) includes standardized follow-up of the children's hips to identify who is at risk of hip displacement. In this study, all children from 4 locations in Sweden with Gross Motor Function Classification System (GMFCS) levels III–V, a Migration Percentage (MP) of <40 % of both hips at the initial radiographic examination, and who had been followed at a minimum of 5 years were included.

We assessed how the HSA predicted the risk of developing a hip displacement with MP >40 % of either hip within 5 years after the first radiographic examination using multiple logistic regressions (corrected for GMFCS level, age and MP). Odds ratios (OR) and CIs are presented.
*Results*: One-hundred and forty-five children (73 boys) fulfilled the inclusion criteria and were included in the study. Of these, 51 developed a hip displacement with MP >40 % during the 5-year follow up and 94 had a MP below 40 % for at least 5 years. The HSA was related to the risk of developing a hip displacement defined as MP >40 %. The OR for one degree increased HSA was 1.07 (95 % CI 1.01–1.14, p = 0.028). This means that when comparing 2 children with the same GMFCS level, age and MP in the most displaced hip, but with a 15 degree difference in HSA, the child with higher HSA has $1.07^{15}$ (1.07 raised in 15) = 3 times higher risk of hip displacement.
*Conclusions*: This study shows that HSA has a predictive value for hip displacement in CP. The study is based on the total population of children with CP in a defined area, a prerequisite to be able to generalize the findings to other children with CP in GMFCS levels III-V. The orientation of the physis reflects the direction of growth. It is therefore logical that hips with a higher HSA have an unfavorable growth direction and a higher risk for lateral displacement.
*Significance*: The HSA seems to be a useful predictor for hip displacement in children with CP.

**OP63**/16:30–16:40

### An evaluation of service provision and outcomes of patients undergoing simultaneous multi level surgery (SEMLS) for cerebral palsy in the Sheffield Childrens Hospital, UK

**Joseph Gaynor**

Sheffield Childrens Hospital, Sheffield, UK

**LEVEL 2/Cerebral palsy**
Keywords: Service provision, SEMLS, Cerebral palsy

*Purpose*: To assess the adequacy of service provision for this population of patients with Cerebral palsy undergoing SEMLS from the perspective of the parents or main carers. Aspects of service provision ranged from outpatient assessment and investigation, through inpatient stay, to post operative rehabilitation and follow up.
*Methods*: Two groups of patients were identified from the hospital database, which comprised patients in GMFCS groups 1 to 3 who had a pre- and post-operative gait analysis; and patients in GMFCS groups 4 or 5 who were non-ambulatory.

Each family was contacted by the first author, and responses obtained to a Likert style questionnaire covering specific aspects of their treatment. The questionnaire was conducted face to face whenever possible, or via telephone if the family was located a long way out of area.
*Results*: Of 191 patients assessed for eligibility, 50 met the inclusion criteria. 33 families completed the study. The majority of patients had a positive experience with subjective and objective improvement in outcome. The study highlighted deficiencies in some areas of service provision, notably in postoperative rehabilitation; supportive care in the community with aids and appliances; and in some instances peri-operative pain relief. The pre-operative functional level of the child was also a predictor of parental satisfaction of outcome.

*Conclusions*: The study identified areas of the service provision that could be improved. Measures are being instituted to address these deficiencies, particularly with respect to the logistics surrounding post-operative care and rehabilitation.

*Significance*: The study highlights inadequacies in service provision for a group of children and families with complex and special needs, as seen from the perspective of the parents or main care givers. This perspective is not always immediately apparent to medical professionals, and can inform those responsible for the provision of such services.

**OP64**/16:40–16:50

## Comparison of hamstring lengthening with hamstring lengthening plus transfer for treatment of flexed knee gait in ambulatory patients with cerebral palsy

**Camila B. R. De Mattos**, Michael Sussman, Jing Feng, Patrick (Khanh) Do, Rosemary Pierce, Michael Aiona

Shriners Hospital for Children Portland, Portland, USA

**LEVEL 2/Cerebral palsy**

Keywords: Cerebral palsy, Flexed knee gait, Hamstring lengthening, Hamstring transfer, Gait analysis

*Purpose*: To evaluate the long term difference of hamstring lengthening alone with hamstring lengthening plus transfer in the treatment of flexed knee gait in ambulatory patients with cerebral palsy.

Some authors believe that recurrence of knee flexion could be prevented by transplanting semitendinosus and gracilis instead of lengthening. Additionally, the re-positioning of the semitendinosis proximal to the knee joint instead of lengthening would maintain the hip extensor power.

We hypothesize that:

1. Hamstring surgery improves knee flexion towards normal during gait
2. Hamstring transfer can maintain the hip extension power during walking
3. Recurrence of increased stance phase knee flexion following hamstring transfer plus lengthening is less compared to the hamstring lengthening

*Methods*: We retrospectively reviewed the gait analysis and physical examinations of 50 patients with ambulatory cerebral palsy GMFCS I, II and III that underwent surgery for flexed knee gait. Thirty-two patients underwent lengthening of the semimembranous ± biceps femoris and transfer of the semitendinous and gracilis to the adductor tendon just proximal to adductor tubercle. Eighteen patients underwent lengthening of the semimembranous, gracilis, semitendinous ± biceps femoris.

All patients underwent pre-operative, 1 year and long term (3.5–5 years) gait analysis and thorough physical exam performed by a physical therapist.

Inclusion criteria: ambulatory patients with cerebral palsy with knee flexion = 20° during the stance phase or popliteal angle = 45°, complete gait studies.

Exclusion criteria: concomitant or subsequent femoral bony surgery, history of selective dorsal rhizotomy, significant athetosis, concomitant Iliopsoas lengthening.

*Results*: The mean age at surgery was 9.9 years. There were 15 patients GMFCS I, 25 GMFCS II and 10 GMFCS III. The follow-up time ranged from 3.5 to 5 years.

On analysis of the physical exam, all patients had a significant ($p < 0.05$) improvement of the Straight Leg Raise, Knee Flexion and Popliteal Angle in both HST and HSL groups in the 1 year post-op.

On analysis of the kinematics, all patients had a significant ($p < 0.05$) improvement on Average Pelvic Tilt and Minimum Knee Flexion in stance in both HST and HSL groups in the 1 year post-op. There was no significant difference on Minimum Hip Flexion in Stance in both HST and HSL groups in the 1 year post-op.

On analysis of the kinetics, there was significant ($p < 0.05$) improvement in the Peak Hip Power in Stance only in the HST group in the 1 year post-op.

On analysis of the physical exam, all patients had a significant ($p < 0.05$) improvement of the Knee Flexion and Popliteal Angle in both HST and HSL groups in the long term. There has no significant difference in improvement in the Straight Leg Raise on both groups in the long-term.

On analysis of the kinematics, there was a significant ($p < 0.05$) improvement on Minimum Knee Flexion in stance in both HST and HSL groups in the long term. There was significant ($p < 0.05$) improvement of the Average Pelvic Tilt only in the HST group in the long term. There was no significant difference in improvement Minimum Hip Flexion in Stance in both groups in the long term.

On analysis of the kinetics, there was significant ($p < 0.05$) improvement in the Peak Hip Power in Stance only in the HST group in the long term.

As complications we had 2 patients that had recurvatum in the post-operative period. There were no infections or nerve palsies.

*Conclusions*: There is no clear benefit from hamstring lengthening and transfer in comparison to hamstring lengthening alone in regards to recurrence at 3.5–5 years of follow-up. In both procedures there was maintenance of stance phase knee flexion. The study shows that there seems to be a continuing improvement of hip extension power in patients that underwent hamstrings lengthening plus transfer. We believe that longer follow-up is needed for additional recurrence information, and also to observe if hip power continues to strengthen with time. At this point there is no clear, outcome defined benefit of the transfer procedure.
*Significance*: This study helps paediatric orthopaedic surgeons to choose between 2 different techniques to treat flexed knee gait in patients with cerebral palsy by showing the long term assessment of gait analysis and thorough physical exam of both procedures.

**OP65**/16:50–17:00

**Spatio-temporal gait analysis in children with cerebral palsy using foot worn inertial sensors**

**Aline Bregou Bourgeois**[1], Christopher Newman[1], Pierre-Yves Zambelli[1], Benoit Mariani[2], Kamiar Aminian[2]

[1]CHUV, Lausanne, Switzerland; [2]EPFL, Lausanne, Switzerland

**LEVEL 4/Cerebral palsy**
Keywords: Gait analysis, Inertial sensors, Cerebral palsy, Child

*Purpose*: Generally, spatio-temporal gait analysis requires dedicated laboratories with complex systems such as optical motion capture. It is possible that a child's natural gait pattern may be affected by a short distance walkway and the laboratory setting. Recently, ambulatory devices have overcome some of these limitations by using body-worn sensors measuring and analyzing gait kinematics.

The aim of this study was to explore the use of foot-worn inertial sensors as a 3D gait measurement tool during a 200-m walking test in independently walking children with cerebral palsy (CP).
*Methods*: We performed a case–control study. We analysed 14 children with CP, aged 6–15 years old, who were followed in our tertiary outpatient child neuro-rehabilitation Unit and 15 controls. There were no significant differences in age (CP11.4 ± 3.6 years, controls 10.6 ± 2.6 years) or gender between cases and controls. In the CP group 9 children were graded GMFCS I, 5 were graded GMFCS II, 12 children had unilateral and 2 had bilateral CP. Two U-shaped and two 8-shaped trial walks per subject were performed during which the accuracy and precision of the foot-worn device was measured using an optical motion capture system (Vicon, Oxford Metrics) as the reference system. All subjects then performed a continuous 200-m walking test at their self-selected pace wearing the foot-worn inertial sensors (Physilog III, LMAM-EPFL, Switzerland). Limb-related spatio-temporal parameters were compared between paretic and control limbs while bilateral gait characteristics were compared between CP and control subjects, using nonparametric analyses.
*Results*: Mean accuracy ± precision for both groups was 3.4 ± 4.6 cm for stride length, 4.3 ± 4.2 cm/s for stride velocity and 0.5 ± 2.9°for initial contact foot pitch angle. For temporal parameters paretic limbs showed longer stance (61.9 ± 2.5 vs 60 ± 0.9 %, $P = 0.006$) and shorter swing (38.1 ± 2.5 vs 39.9 ± 0.9 %, $P = 0.006$) phases, with an increase in double support in children with CP (24.8 ± 4.7 vs 20.3 ± 1.7 %, $P = 0.001$). For spatial parameters stride length (1.07 ± 0.18 m vs 1.32 ± 0.14, $P < 0.001$), speed (1.13 ± 0.23 vs 1.39 ± 0.11 m/s, $P < 0.001$) and peak angular velocity during swing (385 ± 74°/s vs 450 ± 41°/s, $P < 0.001$) were decreased in paretic limbs, with significant differences in foot pitch at both heelstrike and toe-off ($P < 0.001$). Both maximal heel clearance (22.7 ± 3.1 vs 25.6 ± 3.5 cm, $P = 0.004$) and maximal toe clearance (7.6 ± 2.9 vs 13.4 ± 1,6 cm, $P < 0.001$) were lower in paretic limbs.
*Conclusions*: Foot-worn inertial sensors allowed us to analyze gait kinematics outside a laboratory environment with a good accuracy and precision. The case control comparison yielded results, which were congruent with what is known of gait variations in children with cerebral palsy who walk independently. Participants found the system light weight and easy to wear and use.
*Significance*: While not substituting for complete 3D gait analysis, portable sensors provide precise information about gait in conditions that are closer to the child's habitual environment and motor behaviour, and could therefore prove to be a useful complement.

**OP66**/17:00–17:10

**Recurrence of equinus foot deformity after tendo-achilles lengthening in patients with cerebral palsy**

**Ki Hyuk Sung**[1], Chin Youb Chung[2], Kyoung Min Lee[2], Soon-Sun Kwon[2], In Hyeok Lee[2], Sang Hyeong Lee[3], Dae Gyu Kwon[4], Moon Seok Park[2]

[1]Kwandong University Myongji Hospital, Go Yang, Korea; [2]Seoul National University Bundang Hospital, Seong Nam, Korea; [3]Dongguk University Ilsan Hospital, Go Yang, Korea; [4]In-Ha University Hospital, In Cheon, Korea

**LEVEL 4/Cerebral palsy**
Keywords: Equinus deformity, Tendo-Achilles lengthening, Cerebral palsy

*Purpose*: Recurrence of equinus deformity after surgery is common in patients with cerebral palsy. This retrospective study was undertaken to estimate the recurrence rate of the equinus deformity after tendo-Achilles lengthening in patients with cerebral palsy and to investigate the risk factors associated with the recurrence.
*Methods*: Three-hundred thirty eight ambulatory patients with cerebral palsy, who underwent tendo-Achilles lengthening for equinus foot deformity since 1995, and had a pre-operative and post-operative 3D gait analysis, were included. Cox proportional hazards model was used to determine the significant contributing factor for the recurrence of equinus foot deformity.
*Results*: The mean patient age at surgery was 8.1 ± 3.2 years (range 4.4–19.8) and the mean follow-up duration was 6.2 ± 4.2 years (range 0.7–16.7). There was a recurrence in 23 patients (6.8 %) and the Kaplan–Meier survival estimate was shown to be 89.3 % at 10 years not needing repeat surgery. According to the multivariate analysis using the Cox proportional hazard model, pre-operative ankle dorsiflexion at initial contact ($p = 0.023$) was the only significant factor for recurrence of the equinus deformity after surgery. Age at surgery and the type of limb involvement were not associated with the recurrence ($p = 0.328$ and 0.154). The cut off values of preoperative gait kinematics between the non-recurrence and recurrence groups were −19° of ankle dorsiflexion at initial contact ($p = 0.018$).
*Conclusions*: This study showed that the severity of pre-operative equinus deformity was a risk factor associated with recurrence after tendo-Achilles lengthening in patients with cerebral palsy. Therefore, surgeons should consider the recurrence and later revision surgery for the patients with severe equinus foot deformity.
*Significance*: When surgeons treat recurrent equinus deformity after tendo-Achilles lengthening in patients with cerebral plasy, they should take into consideration the fact that pre-operative severe equinus foot deformity is the factor leading to recurrence.

**OP67**/17:10–17:20

## Optimum study design for single-event multilevel surgery in cerebral palsy

**Erich Rutz**[1], Pam Thomason[2], H. Kerr Graham[2]

[1]Murdoch Childrens Research Institute, Melbourne, Australia; [2]Hugh Williamson Gait Laboratory, RCH, Melbourne, Melbourne, Australia

**LEVEL 4/Cerebral palsy**
Keywords: Cerebral palsy, Single-event multilevel surgery, Study design, Outcome

*Purpose*: Levels of evidence are conventionally based on the study design with RCT's accorded Level I status. However, complex surgical interventions may be more amenable to alternative designs, including prospective cohort studies, with objective outcome measures.
*Methods*: The outcomes of two single-event multilevel surgery (SEMLS) studies from a single centre were compared. Study 1 was a RCT and Study 2 was a cohort study, with defined inclusion and exclusion criteria. The primary outcome measure was the Gait Profile Score (GPS) a summary statistic of gait, which incorporates the changes found in 9 kinematic parameters.
*Results*: The study populations in Study 1 and 2 were very similar in terms of baseline characteristics, including GPS (Study 1 mean baseline GPS = 14.9° vs Study 2 = 15.5°). The mean improvement after SEMLS was also similar: Study 1 = 5.5° vs Study 2 = 4.3°. The improvement in both studies was clinically and statistically significant ($p < 0.001$ and more than 3 times the Minimal Clinically Important Difference).
*Conclusions*: No difference was found in the degree of improvement after SEMLS according to study design. The cohort study did not over-estimate treatment effects when compared to the RCT.
*Significance*: RCT's have a limited role in reporting the effects of SEMLS. Prospective, multicentre cohort studies with objective outcome measures and long term follow up may be superior study design.

**OP68**/17:20–17:30

## Ilizarov complex foot deformity correction: a long term follow up

**Hatem Ahmed Kotb**

Limb Lengthening and Bone Deformity Correction Center, Saridar Clinics, Giza, Egypt

**LEVEL 4/Limb reconstruction**
Keywords: Ilizarov complex, Foot deformity, Correction, Long term follow up

*Purpose*: The Ilizarov Method is a known tool in the correction of complex foot deformities. Functional outcome and mobility of the ankle and subtalar joints has been a concern. We report the results of a long term follow up of Ilizarov frame correction for complex foot deformities.
*Methods*: A highly constrained Ilizarov frame was used to correct 55 deformed feet in 41 patients. 27 were males. Mean age at operation was 11.3 + 6.4 years. The preoperative diagnosis included; relapsed and neglected idiopathic congenital talipes equino-varus, paralytic feet, rocker bottom feet, deformed feet associated with syndromes, fibular and tibial hemiamelias, and post trauma and burn deformities. 39 feet had previous operations average 1.9 operations per foot.

A single tibial ring fixation was used in 49 cases. 5 cases were treated by distraction osteotomies and 50 by soft tissue distraction. 9 cases had additional surgical procedures. Clinical, functional and radiographic evaluation was performed. Results were graded according to Ognesyan et al., which includes classification, function; mobility, walking distance, coping with peers, one leg stance and hopping were evaluated.

*Results*: The mean follow up was 11.3 + 3.5 years. The mean fixation time was 2.9 + 1.1 months. All feet were initially corrected except two (frames was removed upon the request of parents and patient). At last follow up 41 (74.5 %) had good results, 6 (10.9 %) had satisfactory results and 8 (14.6 %) had unsatisfactory results. There has been an increase in mobility, full walking distance, one leg stance, hopping and coping with peers. The most common complication was pin track infection, there were no major complications, and no complications related to single tibial ring fixation.

*Conclusions*: Ilizarov fixator is an effective tool for correction of deformed feet. An increase in function can be expected. An increase in the functional and aesthetic results can be anticipated with Ilizarov complex foot correction in the long term.

*Significance*: There is a significant increase in most of functions (increase in mobility, full walking distance, one leg stance, hopping and coping with peers) with Ilizarov complex foot correction.

**April 5th**
Other/Varia
08:40–09:30
CONCERT HALL
**OP69**/08:40–08:50

### The delay of surgical debridement in paediatric open fractures: meta-analysis and systematic review

**Talal Ibrahim**[1], Abdussalam Hijazi[1], Mohammed Riaz[2], Imad Tleyjeh[3]

[1]Hamad Medical Corporation, Doha, Qatar; [2]St George University, London, UK; [3]King Fahad Medical City, Riyadh, Saudi Arabia

**LEVEL 3/Other**
Keywords: Open fractures, Surgical debridement, Infection

*Purpose*: Open fractures are considered orthopaedic emergencies that are traditionally treated with surgical debridement within 6 h after injury. The aim of our study was to investigate the effectiveness of early (less than 6 h from the time of injury) compared to late (greater than 6 h from the time of injury) surgical debridement of paediatric open fractures through a meta-analysis of level I to III studies.

*Methods*: We searched several databases from 1990 to 2013 for level I to III studies that evaluated early and late surgical debridement of pediatric open fractures. We based the main meta-analysis comparison using a random effects model on the odds ratio of infection rate between children undergoing early versus late surgical debridement. We also investigated the infection rate in upper and lower limb open fractures. Descriptive, quantitative and qualitative data were extracted.

*Results*: Of the 98 articles identified, three studies (retrospective cohort studies) were eligible for the meta-analysis, with a total of 714 open fractures. The pooled odds ratio (OR = 0.79) of infection between the early and late surgical debridement groups was in favour of late surgical debridement but not statistically significant (95 % CI 0.32, 1.99; $p = 0.38$, $I^2 = 0$ %). No significant difference in the infection rate was detected between open fractures in the upper and lower limbs according to the time threshold in the included studies (OR = 0.72, 95 % CI 0.29, 1.82; $p = 0.40$, $I^2 = 0$ %).

*Conclusions*: Late surgical debridement was favoured in terms of infection rate in our meta-analysis but this did not reach statistical significance. The cumulative evidence at present does not indicate an association between late surgical debridement and higher infection rates in paediatric open fractures. However, initial expedient surgical debridement of open fractures in children should always remain the rule.

*Significance*: On the basis of this meta-analysis, the classical “six-hour” rule for surgical debridement of pediatric open fractures has little support in the available literature. Thus, multi-center prospective cohort studies will be able to answer this question with more certainty and a higher level of evidence.

**OP70**/08:50–09:00

### Epidemiological analysis of the prevalence and methods of surgical treatment of fractures of the long bones in childhood

**Dorota Hanf-Osetek**, Slawomir Snela, Grzegorz Sokól, Pawel Jasinski

Department of Pediatric Orthopaedics and Traumatology, County Hospital no. 2, Rzeszów, Poland

**LEVEL 2/Other**

Keywords: Fractures of long bones, Epidemiology of long bones fractures, Surgical treatment of long bones fractures

*Purpose*: A retrospective analysis of the epidemiology and methods of treatment of fractures of the long bones in children and adolescents up to 18 years of age treated in the years 2000–2012 in one department dealing with musculoskeletal trauma of childhood.
*Methods*: Analysis of 6,853 fractures in 5,080 patients aged 0–17 years treated in one Department of Paediatric Orthopaedics and Traumatology. In a spreadsheet 12 features were evaluated. Statistical relations were tested using the Chi squared test of independence. The level of significance was set at $p < 0.05$. Due to the accepted standards of treatment, patients were divided into three age categories: 0–3, 4–10, 11–17.
*Results*: Occurrence of statistically significant correlations in the analyzed data: male dominance 69.1 %, a gradual increase in the proportion of male in the following age groups 0–3 years 59.1 %, 4–10 years 64 %, 11–17 years of 74.6 %, a higher incidence of fractures in the summer, from May to August—54.5 %, lower in the winter, from November to February—16 %. Commonest fractures of radius 46.2 %, while in the group of 0–3 years—humerus 40.9 % ($p = 0.00$). The share of the radius fractures was lowest in January and February 29–30 %, the highest between May and September 50–51 %. The share of fractures of the ulna was two times higher in summer than in winter. Fractures of the tibia during the winter months were relatively more frequent. The rate of open reduction decreased and was no more than 10 % ($p = 0.00$). Fractures of diaphyses of the bones of the forearm were more often stabilized using elastic stable intermedullary nails (ESIN) 43 vs.72 % ($p = 0.00$), less frequently we didn't use any internal fixation 53 vs.28 % ($p = 0,00$). Fractures of distal ends of the forearm bones, were more likely to be treated with percutaneous K-wire fixation 14 vs. 50 % ($p = 0.00$), less likely we chose reposition without internal fixation 86 vs. 47 % ($p = 0.00$). In isolated fractures of the distal radius, in more than 80 % we did not use internal fixation. Fractures of the proximal humerus, more often were stabilized with K-wires 33 vs. 60 % ($p = 0.00$), whereas there was no internal fixation in 67 vs. 0 % ($p = 0.00$). In Fractures of the tibial shaft, we used intramedullary fixation in 27 vs. 64 % ($p = 0.00$), much less we used no internal fixation 4 vs. 73 % ($p = 0.00$).
*Conclusions*: Epidemiological analysis of patients treated in one department by one medical team, according to uniform criteria, allows us to establish relationships that influenced the determination of the optimal therapeutic strategy.
*Significance*: The importance in clinical practice in the selection of therapeutic strategies for fractures of long bones in children and adolescents.

**OP71**/09:00–09:10

## An anatomical and radiological study of the distal tibial epiphysis

**Cynthia Van Nguyen**[1], Janelle Dee Greene[1], Daniel Roy Cooperman[2], Raymond Weiwen Liu[1]

[1]Case Western Reserve University, Cleveland, USA; [2]Yale University, Cleveland, USA

**LEVEL 3/Other**

Keywords: Distal tibia, Epiphysis, Anatomy

*Purpose*: In order to guide screw placement about the distal tibial physis, we investigated the topographical anatomy of the distal tibial epiphysis and explored the ability of standard radiographs to visualize physeal undulations.
*Methods*: We studied 30 cadaveric distal tibial epiphyses in specimens 3–14 years of age. AP and lateral radiographs were obtained with and without markers placed on the major undulations and analysed the height or depth of each landmark. In nine specimens, similar measurements were obtained on high-resolution three-dimensional surface scans.
*Results*: Physical examination of the specimens revealed four distinct physeal undulations that were usually present: an anteromedial peak ("Kump's bump"), a posterolateral peak, an anterior central valley and a posterior central valley. On the 3-D scans, Kump's bump averaged 5.0 mm (range 3.0–6.4 mm), the posterolateral peak 2.4 mm (1.2–5.0 mm), the anterior valley 1.3 mm (undetectable to 3.6 mm) and the posterior valley 0.77 mm (undetectable to 2.7 mm). There was no statistical relationship between age and size of any landmark (P ranging from 0.43 to 0.67). Lateral radiographs with markers correlated with measurements from 3-D scans better than those without markers ($r^2 = 0.43$ vs. $r^2 = 0.07$). For AP radiographs, correlation was better with and without the markers ($r^2 = 0.58$ vs 0.60). There was low correlation between the appearances of the landmarks on AP vs. lateral X-rays ($r^2 = 0.10$) but using markers increased the correlation ($r^2 = 0.44$).
*Conclusions*: There are 4 major undulations of the distal tibial physis, with Kump's bump the largest, and their absolute sizes do not correlate with age. A centrally placed epiphyseal screw in the medial/lateral direction or a screw from anterolateral to posteromedial would avoid both valleys. Lateral radiographs varied more from 3-D anatomical scans than AP radiographs, and radiodense markers were necessary on the lateral view accurately to view the undulations.
*Significance*: This study provides quantitative data on the topography of the distal tibial physis to aid in screw placement. Lateral views should be interpreted with caution, as the physeal undulations are not as visible, while AP views can be interpreted with more confidence.

**OP72**/09:10–09:20

### Birth fractures: a 21st century perspective

**Prakash Promod**, Andreas Rehm

Cambridge University Hospitals NHS Trust, Cambridge, UK

**LEVEL 2/Other**
Keyword: Birth fracture

*Purpose*: Fractures during birth are an uncommon but significant event in the perinatal period. The aim of this study was to establish the current incidence of birth fractures and evaluate associated factors.
*Methods*: Out of a cohort of 67,392 deliveries performed over a 12 year period (from 2000–2012) at our University Teaching Hospital, we reviewed all radiographs of patients under the age of 1 year where a fracture had been reported. We looked at the incidence, type of fracture and association with birth weight, singleton and multiple births, gestational age, type of delivery and use of instrumentation. Delays in diagnosis were also investigated.
*Results*: Of the 242 fractures identified in patients under the age of 1 year, 39 were birth fractures, giving an incidence of 3.25 birth fractures annually (26 clavicle fractures, 9 humeral fractures, 2 femoral fractures and 2 parietal bone fractures). All fractures occurred in singletons. 24.8 % of patients were delivered by caesarean section but only 15.4 % of birth fractures occurred during caesarean deliveries. During 18 deliveries with fractures, either ventouse (8) or forceps (10) were used out of 8,982 ventouse and forceps deliveries. The birth weight ranged from 760 to 4,670 g (mean 3,727 g, median 3,890 g) and gestational age from 21 to 42 weeks (mean 38.7 weeks, median 40 weeks). 28 fractures were diagnosed in hospital, 9 within 4 weeks and 2 within 3 months of birth.
*Conclusions*: Birth fractures in our series had a low incidence of 0.58 per 1,000 deliveries despite a large cohort of difficult deliveries with complex medical problems. This fracture rate is far less than previous reports in the literature. We found a clinically significant association between birth fractures and high birth weight. We found no association between birth fractures and caesarean sections, gestational age, multiple births and the use of forceps or ventouse.
*Significance*: Our study shows that the incidence of birth fractures can be much lower as previously reported in a tertiary maternity unit which manages many deliveries of complicated pregnancies, babies with complex problems and a large rate of multiple births (about 200 per year). This supports the establishment of bigger maternity units amongst other reasons to have more expertise available to reduce birth trauma in children. Our data also provide a basis which helps to differentiate between birth fractures and non-accidental fractures occurring very early in life.

**OP73**/09:20–09:30

### Complex regional pain syndrome type 1 in children

**X. Bayle Iniguez**, C. Pajot Audouin, J. Sales De Gauzy, F. Accadbled

Hôpital des Enfants, Toulouse, France

**LEVEL 4/Other**
Keywords: Complex regional pain syndrome, Allodynia, Ankle, Quality of life

*Purpose*: Complex regional pain syndrome type 1 (CRPS1) in children differs from its adult counterpart. Literature is scarce about the diagnosis, evolution and outcome. The aim of the study was to describe this condition and to assess it's midterm outcome.
*Methods*: Medical records of patients diagnosed with CRPS1 between 2004 and 2012 at a single institution were reviewed and analysed. Patients and parents were called for a phone interview including PEDS Quality of Life 4-0 questionnaire (score out of 100). Results were then compared to those obtained from a matching control group.
*Results*: 73 patients were included (64 girls, 9 boys). Medical history showed 52 % atopic profiles and 42 % somatoform disorders (abdominal pain or neurological manifestations). A history of psychological care was noted in 30 % of cases and speech therapy in 27 % of cases. Patients were rated as good or very good students in 92 % of cases, perfectionist in 63 % of cases and anxious in 90 % of cases. 63 % were reported to have sleep disorders. The mean age at diagnosis was 11.4 years, and the mean time between onset of symptoms and diagnosis was 14 months. 36 patients reported a physical injury (minor in 33 cases) at least one psychological event was identified in 37 patients. The lower limb was affected in 89 % of cases (83 % foot and ankle) and the upper limb in 11 % of cases (at the wrist exclusively). 44 % of the patients had multiple localizations, of which 25 % were bilateral. Clinical presentation always included complete loss of function of the affected limb. Allodynia was noted in 95 % of cases, coldness in 81 % of cases, and cyanosis in 74 % of cases. 42 MRI scans were performed. 11 (26 %) were in favour of CPR1. 25 bone scans were performed, with 11 (44 %) showed hypofixation. At a mean 36 months follow up, 44 patients out of 50 having at least 12 month follow up were reached by phone. 85 % of them acknowledged healing or marked improvement and 57 % presented a recurrence of symptoms. On average, CRPS1 patients had a poorer PEDS QL score than matching controls (77 vs 88, $p = 0.02$). The 29 CPRS1 patients managed with psychological support (69 %) had a poorer PEDS QL 4.0 score than those without it (71 vs 88, $p = 0.0032$).
*Conclusions*: The results of this study are consistent with the scarce literature. We emphasized the uncertain and relatively poor outcome together with the large psychological component of this condition. The fact that patients with psychological care had poorer results than those without it came as a surprise. This may be the result of their more vulnerable personality.
*Significance*: CRPS1 in children is poorly described and too often misdiagnosed and late diagnosed. Imaging rather than plain radiographs are more useful in ruling out the differential diagnoses.

**April 5th**
Upper Extremity Trauma and Tumors
11:25–12:15
CONCERT HALL
**OP74**/11:25–11:35

### The effect of social deprivation on paediatric fractures

**Rishikesan Ramaesh**, Nick Clements, Louise Rennie, Mark S. Gaston

Royal Hospital for Sick Children, Edinburgh, UK

**LEVEL 3/Trauma—Upper limb**
Keywords: Social deprivation, Fractures, Children, Incidence, Epidemiology

*Purpose*: Childhood trauma is common and fractures account for 10–25 % of all injuries sustained. Paediatric fractures are a source of morbidity in childhood and can result in long term disability. Social deprivation has been associated with an increased incidence of fractures in both adult and adolescent populations. No study however has examined the effect of social deprivation on incidence of fractures in childhood.
*Methods*: All paediatric fractures (aged less than 16) from three county districts in the South East of Scotland presenting to one hospital were collected between January 2000 and December 2000 using a prospective database. Records were retrospectively analysed, and the X-rays checked by an independent reviewer to confirm the fracture. Data was collated on epidemiology, type of fracture, mode of injury and post-code. Deprivation scores were calculated using the Scottish Index for Multiple Deprivation (SIMD) and patients were divided into quintiles based on the SIMD score. Spearman's correlation was used to calculate the correlation between quintiles and odds ratios between the most affluent and least affluent and significance was calculated.
*Results*: There were a total of 2,195 consecutive fractures presenting over a 12-month period. There was a significant correlation of deprivation with incidence of fractures ($p = 0.0083$, $r = 1.00$)—the most deprived children had rates of 2420/100,000/year compared with the least deprived at 1,775/100,000/year. Deprivation was associated with an increased risk in most types of fractures including distal humerus, metacarpals and clavicle fractures. The mode of injury also showed variations between the most and least deprived. The poorest fifth of children are more likely to suffer injuries as a result of falls ($OR = 1.5$, $p < 0.0001$), blunt trauma ($OR = 1.5$, $p = 0.026$) and road traffic accidents ($OR = 2.7$, $p < 0.0001$) compared to the most wealthy fifth.
*Conclusions*: This study is the first to show that social deprivation is a significant risk factor for sustaining a fracture in childhood. We show that less well off children are more likely to sustain a fracture and a pattern of mechanisms by which those fractures are sustained. For example, the increased incidence of fractures through falls in the more deprived could be explained by the decreased availability of safety equipment and poor design of social housing or reduced supervision, while the increase in road traffic accidents may be due to inadequate access to safe-play areas or insufficient traffic calming measures in these deprived areas. These findings have important implications for public health interventions and preventative measures, to reduce the overall fracture morbidity and load.
*Significance*: Fractures occur more commonly in children from deprived areas and focusing public health interventions towards preventative measures in these areas will help to lower the rate of injury in deprived children.

**OP75**/11:35–11:45

### Remodeling of severe residual angulation of distal radius fractures in children: quantitative data on the speed of remodeling

**Kimberly Jeroense**, Fleur Joor, Christien Sleeboom, Melinda Witbreuk

VUmc, Amsterdam, The Netherlands

**LEVEL 2/Trauma—Upper limb**

Keywords: Remodeling, Angulation, Distal radius fractures, Remodeling speed

*Purpose*: Paediatric forearm fractures have excellent remodeling potential, but to what extent conservative management is justified in case of residual angulation, is up for debate. In the current literature 15° is often mentioned as maximum angulation boundary, though studies that focus on remodeling capacity above this value are lacking.

Therefore, the objective of this retrospective study is for the first time to present data on remodeling speed in children with conservatively managed distal radius fractures with residual angulation of 15° or more.

*Methods*: Retrospectively we evaluated the angular remodeling in 33 children (aged 3–14 years), with a total of 40 wrists fractures healed with residual angulation of ≥15° in the dorso-volar (DV, n = 32) and/or the radio-ulnar (RU, n = 8) plane. Fracture angulation and residual angulation at the time of the last follow up (FU) were measured on radiographs of the fracture site on AP and lateral views. We chose a maximum follow up time of 30 months.

*Results*: All fractures showed remodeling after a mean FU of 8 months (2.5–29 months). Mean DV fracture angulation was 23° (15°–49°) and RU 21° (15°–33°). At FU this had remodeled to a mean 8° DV and 10° RU. Mean remodeling speed (RS) was 2.5° per month (0.4°–7.6°). There was no significant difference in speed between the DV and RU planes. The RS was negatively correlated with remodeling time (RT) ($p = 0.001$) and positively correlated with fracture angulation ($p = 0.002$). This relationship appeared to be non linear. Combining both significant variables resulted in a predictive value of 61 % for RS. The RS was not significantly related to age or gender ($p = 0.420$; $p = 0.437$; respectively).

*Conclusions*: The limit for acceptable residual angulation for conservative treatment appears to have been set too cautiously in the current literature. Since we found that greater angulations are correlated with a greater remodeling speed.

*Significance*: Perhaps greater residual angulations could be accepted for conservative therapy, which would eliminate unnecessary risks for the patient and healthcare costs.

**OP76**/11:45–11:55

## Wrist arthroscopy in children and adolescents with chronic wrist pain

**P. Nithin Unnikrishnan**, Ashok Ramavath, J. H. Stillwell, Harvey L. George, Colin Bruce, P. Sathyamoorthy

Royal Liverpool Childrens Hospital (Alder Hey), Liverpool, UK

**LEVEL 3/Trauma—Upper limb**

Keywords: Wrist arthroscopy, Chronic wrist pain

*Purpose*: Wrist arthroscopy is a dynamic diagnostic procedure and its indications are growing as a treatment modality in the adult population. The aim of the study was to report retrospectively our series of paediatric and adolescent patients with chronic wrist pain, with or without wrist instability who underwent wrist arthroscopy after failing at least 4 months of conservative management. Our secondary aim was to report the sensitivity and specificity of clinical examination and MRI evaluation for various injury subgroups against the gold standard of the arthroscopic findings. The technical challenges, complications and outcomes are also discussed.

*Methods*: A retrospective review of the medical records of thirty two paediatric and adolescent patients who underwent wrist arthroscopy was conducted. Pre-operative clinical diagnosis, radiographic and intra-operative findings including classifications of triangular fibro cartilage complex (TFCC) and interosseous ligaments were obtained. Patients were followed up to 1 year post op and were discharged if symptom free.

*Results*: Thirty three wrist arthroscopies in 32 patients were performed from 1996–2004. There were 2 male and 29 female patients. At arthroscopy 17 wrists were found to have TFCC injuries, 11 wrists had SL injuries and 8 had LT injuries (Table 2). Clinical examination for diagnosis of TFCC injury was too sensitive and non specific. However the clinical diagnosis of SL injury was sensitive and specific. MRI was found to have a low sensitivity for diagnosis of LT injury but diagnosis of TFCC was sensitive and specific

*Conclusions*: The gender ratio of 2 male: 29 female was startling. It may have been postulated that some patients in this age range could have been unreliable witnesses but this study does not support this notion because pathology was identified at arthroscopy in 31 of the 33 wrists.

*Significance*: The gender ratio of 2 male: 29 female was startling. It may have been postulated that some patients in this age range could have been unreliable witnesses but this study does not support this notion because pathology was identified at arthroscopy in 31 of the 33 wrists.

**OP77**/11:55–12:05

## Anterior dislocation of the shoulder in skeletally immature patients: comparison between non-operative treatment versus an open Latarjet's procedure

**Federico Canavese**[1], Ahmad Khan[1], Antoine Samba[1], Bruno Pereira[2]

[1]Department of Pediatric Surgery, CHU Estaing, Clermont Ferrand, France; [2]Biostatistics unit, DRCI. CHU Clermont-Ferrand, Clermont Ferrand, France

**LEVEL 3/Upper extremity**
Keywords: Skeletally immature, Shoulder instability, Latarjet's procedure, Dislocation, Children

*Purpose*: Most of the studies published to date on shoulder instability have focused on skeletally mature patients. It is unclear whether it is possible to apply the proposed decision making algorithm to skeletally immature patients.

The aim of the study is to evaluate functional outcome of surgical and non surgical techniques within the skeletally immature group, using QuickDASH scale, as well as to investigate whether the Latarjet's technique is applicable in skeletally immature patients. Long-term functional/radiological outcomes and arthritic gleno-humeral changes after surgery were investigated as well.

*Methods*: Skeletally immature patients less than 16 years old with growth plate visible on plain radiographs, and radiologically proven anterior shoulder dislocation without concomitant fracture, nor neurovascular injuries followed at two institutions (1993–2010), were included. Patients from one institution were never treated surgically (Group I), while patients from the other institution were submitted to the Latarjet's procedure if they had ISIS equal to 5 and at least 3 episodes of dislocation (Group II).

*Statistical analysis*: Tests were two-sided, with a type I error set at $a = 0.05$. Baseline characteristics were presented as mean ± standard deviation (SD) for each gender group (surgical/non-surgical) for continuous data and as the number of patients and associated percentages for categorical parameters. Comparisons between groups were analyzed using Chi squared or Fisher's exact test for categorical variables, and Student's *t* test or Mann–Whitney test for quantitative variables, with normality verified by the Shapiro–Wilk test and homoscedasticity by the Fisher–Snedecor test. For intra-group paired comparisons, paired *t* test or Wilcoxon tests were considered for quantitative parameters (re-dislocation/dislocation and external rotation) and McNemar for binary outcomes (apprehension).

*Results*: Groups were homogenous (gender, age at first dislocation, quantity, mean follow-up). Group I: 23 patients, 25 shoulders; Group II: 26 patients, 28 shoulders.

All patients showed reduction of external rotation ($p < 0.001$). Although Group II patients showed loss of external rotation with means of 62.1° versus 74.8° of Group I ($p = 0.001$), they could return to the same level of sport activities in 92 % of patients versus 52 % of Group I patients.

Group II patients showed a less painful shoulder and better scoring of the QuickDASH questionnaire (5.8 ± 11.2 versus 11.7 ± 21.1 in Group I) ($p = 0.41$).

Apprehension tests were positive in 60 % of shoulders in Group I and 25 % in Group II ($p = 0.01$).

Eight shoulders (32 %) of Group I had Hill-Sachs lesion and 1 (4 %) shoulder had bony Bankart lesion. Four shoulders (14.2 %) of Group II had partial to complete coracoid bone block resorption. There were no gleno-humeral arthritic changes, according to Samilson classification, in either Group I or II. High ISIS scores were associated with increased rate of recurrence.

*Conclusions*: Mid to long-term results are good to excellent in post-surgical patients and fair in non surgically treated patients. Post-surgical patients showed better signs of shoulder stability than others who have a higher rate of recurrence.

The first episode of dislocation in skeletally immature patients should be treated conservatively regardless of age of onset. After one or more recurrences, Latarjet's stabilization can be performed.

*Significance*: We found no contraindications to operate on the skeletally immature patient, especially if they engage in high-energy physical activities, nor in those with existence of Hill-Sachs lesion, as well as hyperlax patients

**OP78**/12:05–12:15

## Evaluation of forearm abnormalities; clinical and cosmetic results of surgical treatment in patients with multiple hereditary exostoses. A retrospective explorative study

**Mark Flipsen**[1], Vanessa Scholtes[1], John Ham[1], Konrad Mader[2]

[1]Onze Lieve Vrouwe Gasthuis, Amsterdam, The Netherlands; [2]Sentralsykehuset, Førde, Norway

**LEVEL 4/Upper extremity**
Keywords: Multiple hereditary, Exostoses, Exostose osteochondroma, Osteochondromas, Forearm, Radial, Ulnar, Lengthening, Correction, Osteotomy

*Purpose*: Forearm osteochondromas and/or deformities are found in a vast majority of patients diagnosed with multiple hereditary exostoses (MHE). Common complaints in this cohort of patients include pain, functional impairment, and cosmetic concerns. So far, there is no consensus regarding indications for surgery or optimal treatment regimens. This study aimed retrospectively to describe and assess the results of surgical treatment for forearm osteochondromas and deformities in a large cohort of MHE patients. Secondary aim was to evaluate the patient's general and cosmetic satisfaction, and quality of life.

*Methods*: 124 forearms in 97 patients were surgically treated for forearm osteochondromas or deformities between 2002 and 2013 and were included in this study. Range of forearm, elbow, and wrist motion, were documented preoperative and at 2, 5, and 10 years after surgery.

Forearm function measurements included supination and pronation; elbow measurements included flexion and extension, wrist measurements included dorsal extension, palmar flexion, radial and ulnar deviation. A cross-sectional questionnaire was sent to all 97 patients. The questionnaire focused on patient general and cosmetic satisfaction, pre- and postoperative pain, and quality of life. Patients were grouped by indication for surgery (i.e. pain, functional impairment, or both). Results were statistically analyzed with use of the SPSS 15.0 software and with the Chi-square test and multiple logistic regression.

*Results*: Seventy-two patients (74 %) completed the questionnaires, including 27 adults (38 %) and 45 children (62 %). The median follow-up period was 41 months (range 7.5–139 months). Surgical procedures performed included excision of forearm osteochondromas, ulnar lengthening, radial corrective osteotomy, excision of radial head, or combinations. On the basis of our findings, the assessment for indication as stated is justifiable. The range of motion in patients with an indication for surgery due to functional impairment was significantly lower compared to patients with other indications ($p = 0.000$ for supination, pronation, and radial deviation). In patients with complaints of pain preoperatively, this significantly decreased after surgery.

*Conclusions*: Significant improvements were made in pain complaints and range of motion by excision of osteochondromas or corrective procedures in patients with forearm deformities in MHE. Cosmetic complaints should not be solely used as an indication for surgery. As the quality of life in MHE patients is clearly affected, forearm involvement has a significant contribution to the comprehensive issues MHE patients face.

*Significance*: Level of Evidence: Level IV

Open Access

The abstracts are distributed under the terms of the Creative Commons Attribution License which permits any use, distribution, and reproduction in any medium, provided the original author(s) and the source are credited.

